# Protective potential of piroxicam on human peripheral blood mononuclear cells against the suppressive capacity of glioblastoma cell lines

**DOI:** 10.1038/s41598-022-24392-2

**Published:** 2022-11-17

**Authors:** Jahangir Abdesheikhi, Farnaz Sedghy, Alireza Farsinejad, Merat Mahmoudi, Mahdi ranjkesh, Meysam Ahmadi-Zeidabadi

**Affiliations:** 1 Department of Immunology, School of Medicine, Kerman University of Medical Sciences, Kerman, Iran; 2 Cell Therapy and Regenerative Medicine Comprehensive Center, Kerman University of Medical Sciences, Kerman, Iran; 3 Department of Hematology and Laboratory Sciences, Faculty of Allied Medical Sciences, Kerman University of Medical Sciences, Kerman, Iran; 4 Institute of Neuropharmacology, Neuroscience Research Center, Kerman University of Medical Sciences, Kerman, Iran; 5 Faculty of Medicine, Shahid Bahonar University, Pajoohesh Sq, Kerman, 7616914111 Iran

**Keywords:** Cancer, Immunology, Neuroscience

## Abstract

Dexamethasone, a common medication used in the treatment regimen of glioblastoma, has broad inhibitory effects on the immune responses. Here, in an in vitro study, we examined the effects of piroxicam, a potent substitute for dexamethasone, on peripheral blood mononuclear cells (PBMCs) co-cultured with two glioblastoma cell lines, U-87 MG and A-172 cells. MTT assay was used to determine the proliferation of PBMCs treated with piroxicam, or dexamethasone. In addition, to evaluate the effects of drugs on the cell cycle distribution, DNA content per cell was analyzed in PBMCs and A-172 cell lines using flow cytometry. Oxidative parameters, including superoxide dismutase-3 (SOD3) activity and total anti-antioxidant capacity, lactate dehydrogenase (LDH) activity, as well as IFN-γ and TGF-β levels were measured in PBMCs alone or in the presence of cell lines using ELISA. Unlike dexamethasone, piroxicam showed a protective effect on PBMCs against both glioblastoma cell lines. Furthermore, while dexamethasone reduced the proliferation of PBMCs, piroxicam had no adverse effect on the proliferation. Cell cycle analysis showed a reduction in the G2/M phase in piroxicam-treated A-172 cells. Additionally, dexamethasone limited the cell cycle progression by increasing the fraction of PBMCs in G0/G1. Interestingly, after co-culturing piroxicam-treated PBMCs with cell lines, a remarkable rise in the LDH activity was observed. Although not significant, piroxicam partially decreased TGF-β levels in both cell lines. Our findings suggested a protective effect of piroxicam, but not dexamethasone, on PBMCs against inhibitory mechanisms of two glioblastoma cell lines, U-87 and A-172 cells.

## Introduction

Glioblastoma multiform (GBM) is considered the most common and invasive malignancy of the central nervous system and is clinically classified into two types, primary and secondary^1,2^. Primary glioblastoma, accounting for 80% of GBM cases, occurs suddenly in older patients (mean age 62 years); and secondary glioblastoma, which develops from low-grade astrocytoma or oligodendroglioma and takes place in younger patients (mean age 45 years)^1,3^. Current standard therapies for glioblastoma include surgery, followed by radiation and chemotherapy. Despite these treatments, the disease correlates with a poor prognosis, with a median life expectancy of 15 months in the patients^4,5^.

Glioblastoma exerts an immense immunosuppressive microenvironment, especially on T cells, the most prominent immune cells providing immunity against tumors. GBM is considered a “cold tumor”, characterized by the lack of effector T cells in the tumor microenvironment. Further, effector functions of T cells are influenced mainly by the presence of various immunosuppressive factors, including TGF-β, and excessive secretion of reactive oxygen species (ROS) by tumor cells. Accumulation of ROS results in T-cell dysfunction and increased apoptosis, while regulatory T (Treg) cells exhibit more resistance in this environment, causing the predominance of Treg cells in the tumor tissue^6^. Besides, the overexpression of immune checkpoints on the surface of T cells, and the inefficiency of antigen presentation to T cells deteriorate the anti-tumor activity of these cells^7^.

Dexamethasone is a potent synthetic glucocorticoid used in glioblastoma treatment regimens to relieve patients' neurological symptoms as well as chemotherapy-induced nausea and vomiting^8,9^. However, Dexamethasone-induced T cell suppression might interfere with antitumor activity. In addition, some evidence implied that dexamethasone administration, particularly in high doses (> 8 mg/kg), reduced survival in animal and human studies^10,11^. Therefore, identifying novel therapeutic agents appears necessary.

Nonsteroidal anti-inflammatory drugs (NSAIDs) are a range of medications prescribed to relieve pain, fever, and inflammation^12,13^. These drugs exert their anti-inflammatory properties by inhibiting the enzyme cyclooxygenase (COX), most importantly the induced isoform of this enzyme, COX-2, and thus inhibiting the synthesis of prostaglandins (PGs)^13^. COX‐2/prostaglandin E2 (PGE2) pathway induction is a major characteristic in cancer cells with immunosuppressive effects within the tumor microenvironment^14^. Some research has shown that the use of NSAIDs is associated with a decreased risk of several types of cancers such as breast^15^, prostate^16^, colorectal^17^, and ovarian^18^ cancers. These anti-cancer effects of NSAIDs are probably due to their anti-inflammatory actions^19^, the ability to induce apoptosis in tumor cells^13^, impair angiogenesis, and enhance T cell-dependent cellular immune responses^20,21^, probably by suppressing PGE2 synthesis. Several mechanisms have been suggested to describe the inhibitory role of PGE2 on T cells. For example, some evidence reported the elevated frequency of regulatory T cells^22^, and reduced anti-tumor activity of CTLs^23,24^ in the presence of PGE2. Also, a positive correlation between the levels of PGE2 in the lung cancer tissue and the expression of PD-1 (an inhibitory checkpoint molecule) on the surface of CD8 + T cells^25^. Additionally, a role has been considered for PGE2 in the differentiation of monocytes to M-MDSC-like cells, important cells in attenuating effector T cell function and promoting regulatory T cell induction^24^. Moreover, inhibition of the COX‐2 enzyme resulted in a depleted amount of TGF‐β in the tumor microenvironment, suggesting a role for this enzyme in regulatory T cell induction, TGF‐β production, and subsequently cancer progression^14^.

Piroxicam is a class of NSAIDs with similar anti-inflammatory properties, which inhibit the COX enzyme and thereby, the production of prostaglandins^26^. The anticancer effects of piroxicam have been indicated in some in vitro studies. Rai et al. in 2015 showed that piroxicam elicited the expression of reactive oxygen species (ROS), and as a result, apoptosis in the human breast cancer cell line^27^. Moreover, the anti-edematous action of this drug has been reported^28,29^, rendering this medication a potent candidate to treat brain cancers.

Here, we designed an in vitro study to evaluate the effects of piroxicam on peripheral blood mononuclear cells (PBMCs) co-cultivated with two glioblastoma cell lines, U-87 malignant glioma (MG) and A-172 cells and to compare these effects with dexamethasone. The main focus of this paper is to examine the viability and metabolic activity, as well as cytokine levels in stimulated PBMCs to explore T cell function in the presence of glioblastoma cell lines.

## Results

### Piroxicam protects PBMCs against the inhibitory effects of U-87 MG and A-172 cell lines

**Figure 1:**
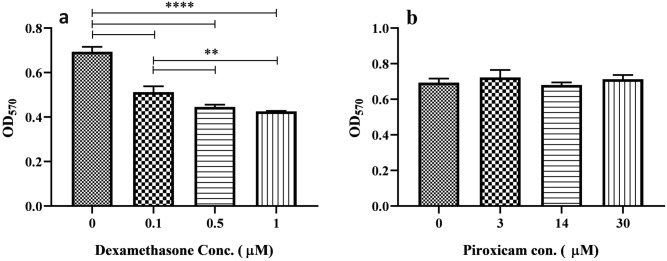
The effect of drugs on the proliferation of T cells in PBMCs. The effect of three different concentrations of dexamethasone (**a**) and piroxicam (**b**) was assessed on the proliferation of T cells in PBMCs (n = 3). The graphs show the mean ± SD. Significant changes are indicated with asterisk (*p < 0.05, **p < 0.01, ***p < 0.001, ****p < 0.0001).

**Figure 2:**
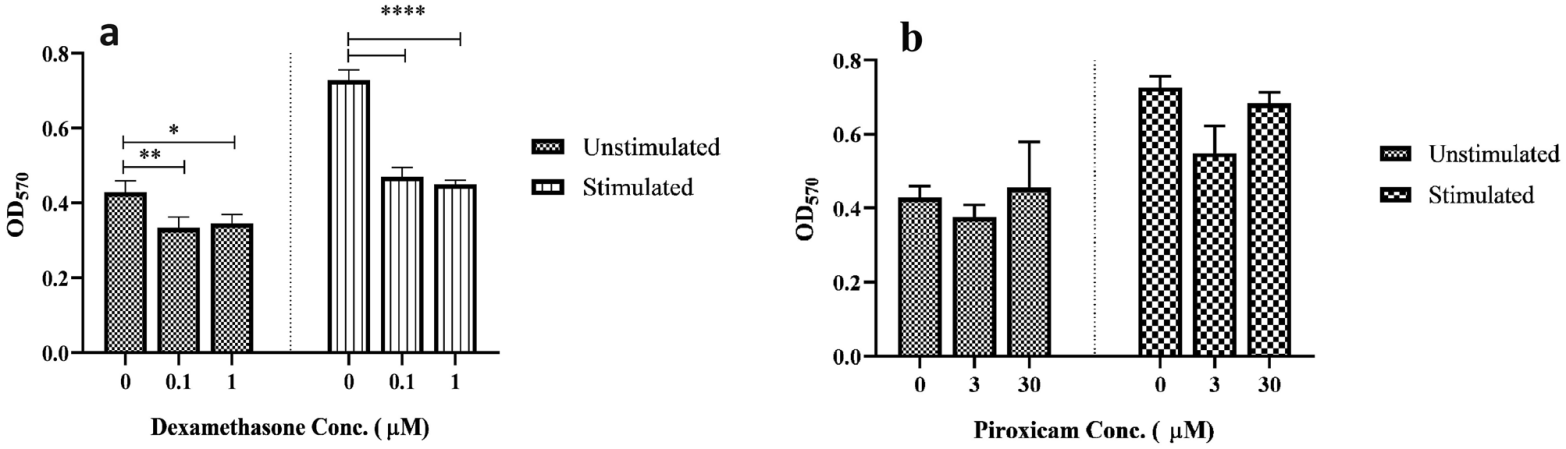
The effect of drugs on the proliferation of T cells in PBMCs in stimulated and non-stimulated conditions. The effect of two different concentrations of dexamethasone (**a**) and piroxicam (**b**) on the proliferation of T cells in PBMCs was compared in stimulated and non-stimulated conditions (n = 3). The graphs show the mean ± SD. Significant changes are indicated with asterisk (*p < 0.05, **p < 0.01, ***p < 0.001, ****p < 0.0001).

To measure proliferation, PBMCs were treated with three different concentrations of piroxicam (3, 14, and 30 µM) and dexamethasone (0.1, 0.5, and 1 µM) for 72 h at 37 °C, 5% CO2, and 95% humidity, and T cells were stimulated with anti-CD3 and anti-CD28 antibodies. Data obtained from MTT [3-(4,5- dimethylthiazol-2-yl)-2,5-diphenyltetrazolium bromide] assay showed that while dexamethasone reduced significantly the proliferation of PBMCs (Fig. 1a, Table 1s, *p* < 0.0001), piroxicam had no adverse effect on the proliferation (Fig. 1b, Table 1s). We further compared PBMC proliferation in stimulated and non-stimulated conditions. To this end, PBMCs were treated with two concentrations of piroxicam (3 and 30 µM) and dexamethasone (0.1 and 1 µM) either in stimulated or non-stimulated conditions. As shown in Fig. 2, PBMC proliferation was notably lower in the stimulated compared to the non-stimulated condition when treated with dexamethasone (Fig. 2a, Table 2s, *p* < 0.0001, and *p* < 0.05 respectively), whereas in piroxicam-treated PBMCs, no difference was observed in proliferation of stimulated and non-stimulated cells (Fig. 2b, Table 2s).

**Figure 3:**
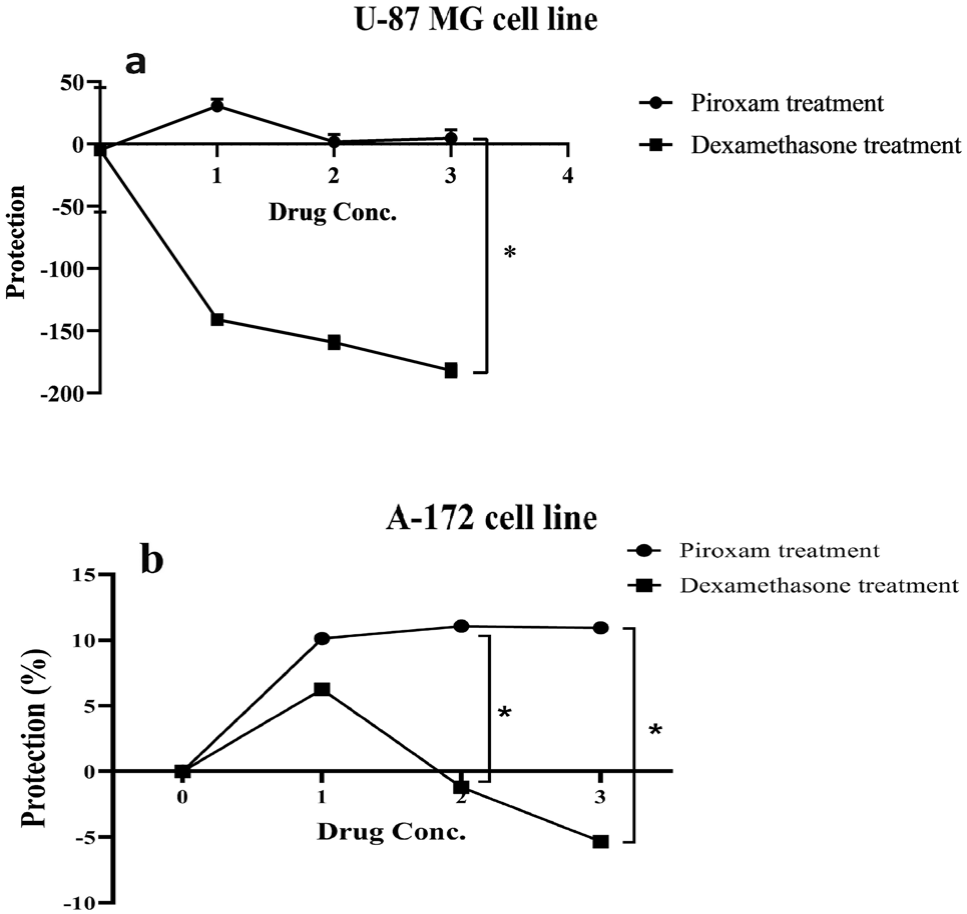
The effect of drugs on the protection of T cells in PBMCs co-cultured with U-87 MG and A-172 cell lines. The effect of three different concentrations of dexamethasone and piroxicam was determined on the protection of T cells in PBMCs cocultured with U-87 MG (**a**) (n = 3), and A-172 (**b**) (n = 4) cell lines. The graphs show the mean ± SD. Significant changes are indicated with asterisk (*p < 0.05, **p < 0.01, ***p < 0.001, ****p < 0.0001).

We examined the effects of piroxicam compared to dexamethasone on the protection of PBMCs in the presence of the cell line. PBMCs were co-cultured with U-87 MG and A-172 cell lines and treated with three different concentrations of piroxicam (3, 14, and 30 µM) and dexamethasone (0.1, 0.5, and 1 µM) for 72 h at 37 °C, 5% CO2, and 95% humidity. Unlike dexamethasone, piroxicam showed a favorable protective effect on PBMCs against both U-87 MG (Fig. 3a, Table 3s, *p* = 0.018) and A-172 (Fig. 3b, Table 4s, *p* = 0.043 in 14 µM, and *p* = 0.018 in 30 µM) cell lines.

### Piroxicam led to a reduction in the G2/M phase in the cell cycle of A-172 line

**Figure 4:**
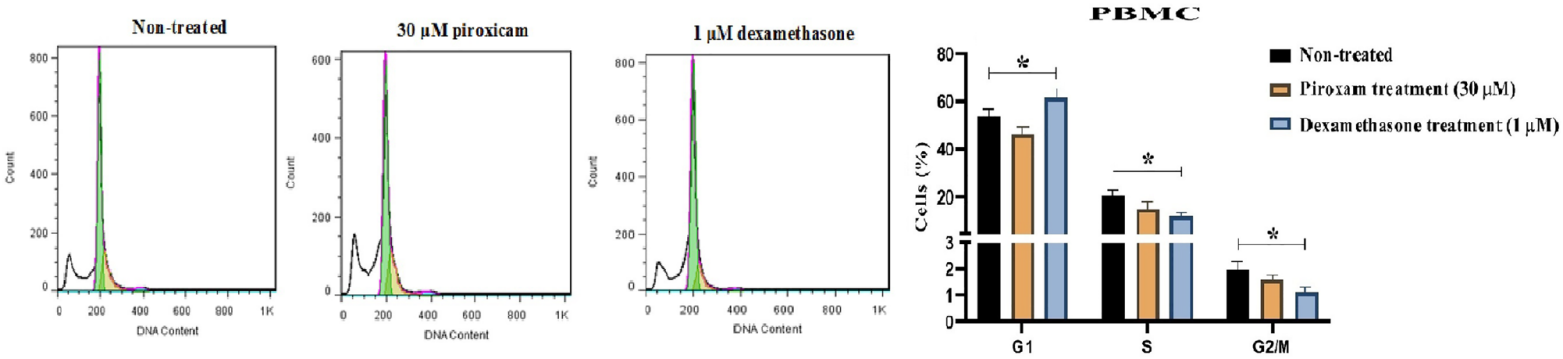
The effect of drugs on the cell cycle phase of T cells in PBMCs. The effect of dexamethasone and piroxicam was measured on the cell cycle phase of T cells in non-treated PBMCs, PBMCs treated with 30 μM piroxicam or with 1 μM dexamethasone (n = 3). The graphs show the mean ± SD. Significant changes are indicated with asterisk (*p < 0.05, **p < 0.01, ***p < 0.001, ****p < 0.0001).

**Figure 5:**
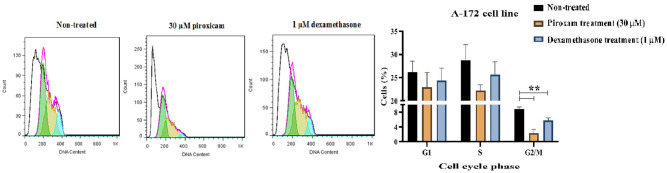
The effect of drugs on the cell cycle phase of A-172 cell line. The effect of dexamethasone and piroxicam was measured on the cell cycle phase of A-172 cells in non-treated, and treated with 30 μM piroxicam or with 1 μM dexamethasone (n = 3). The graphs show the mean ± SD. Significant changes are indicated with asterisk (*p < 0.05, **p < 0.01, ***p < 0.001, ****p < 0.0001).

To evaluate the effects of drugs on the cell cycle distribution, we analyzed the DNA content per cell using PI staining. As shown in Fig. 4, dexamethasone limited the cell cycle progression by significantly increasing the fraction of PBMCs in G0/G1, while the accumulation of cells in the S and G2/M were decreased (Fig. 4, Table 5s, *p* = 0.047 in G0/G1, *p* = 0.015 in S, and *p* = 0.034 in G2/M). Additionally, treatment of A-172 cells with piroxicam led to a reduction in the G2/M phase (Fig. 5, Table 6s, *p* = 0.002), but no considerable change was detected in the G0/G1 and S phases. Similar effects were observed in dexamethasone-treated cell lines (Fig. 5, Table 6s, *p* = 0.012).

### Piroxicam elevated T cell metabolism in the co-culture condition via reducing lactate dehydrogenase (LDH) activity in glioblastoma cell lines

**Figure 6:**
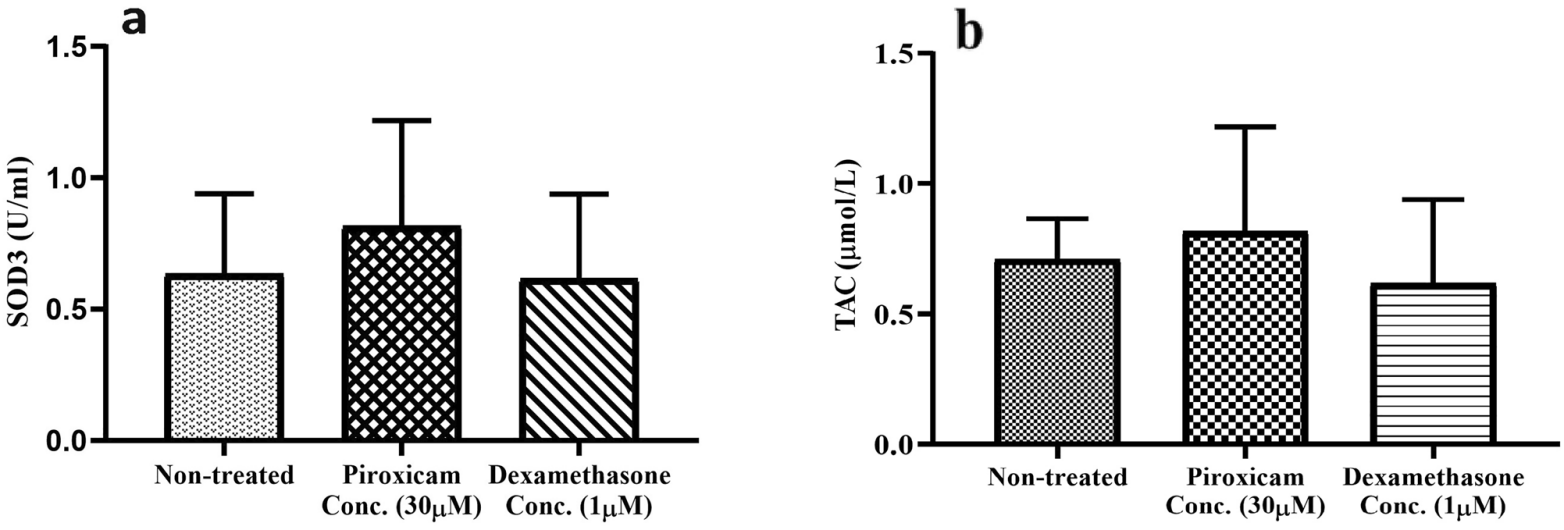
The effect of drugs on the oxidative activity of T cells in PBMCs. The effect of dexamethasone and piroxicam was measured on the SOD3 activity (**a**) and TAC levels (**b**) of T cells in PBMCs (n = 3). The graphs show the mean ± SD. Significant changes are indicated with asterisk (*p < 0.05, **p < 0.01, ***p < 0.001, ****p < 0.0001).

The anti-oxidative activity of PBMCs in the presence of piroxicam and dexamethasone was examined. Cells were treated with 30 µM piroxicam and 1 µM dexamethasone, stimulated with anti-CD3 and anti-CD28 antibodies, and then incubated for 72 h at 37 °C, 5% CO2, and 95% humidity. Superoxide dismutase-3 (SOD3) activity (Fig. 6a, Table 7s) and total antioxidant capacity (TAC) amounts (Fig. 6b, Table 8s) in treated PBMCs were similar to non-treated cells.

**Figure 7:**
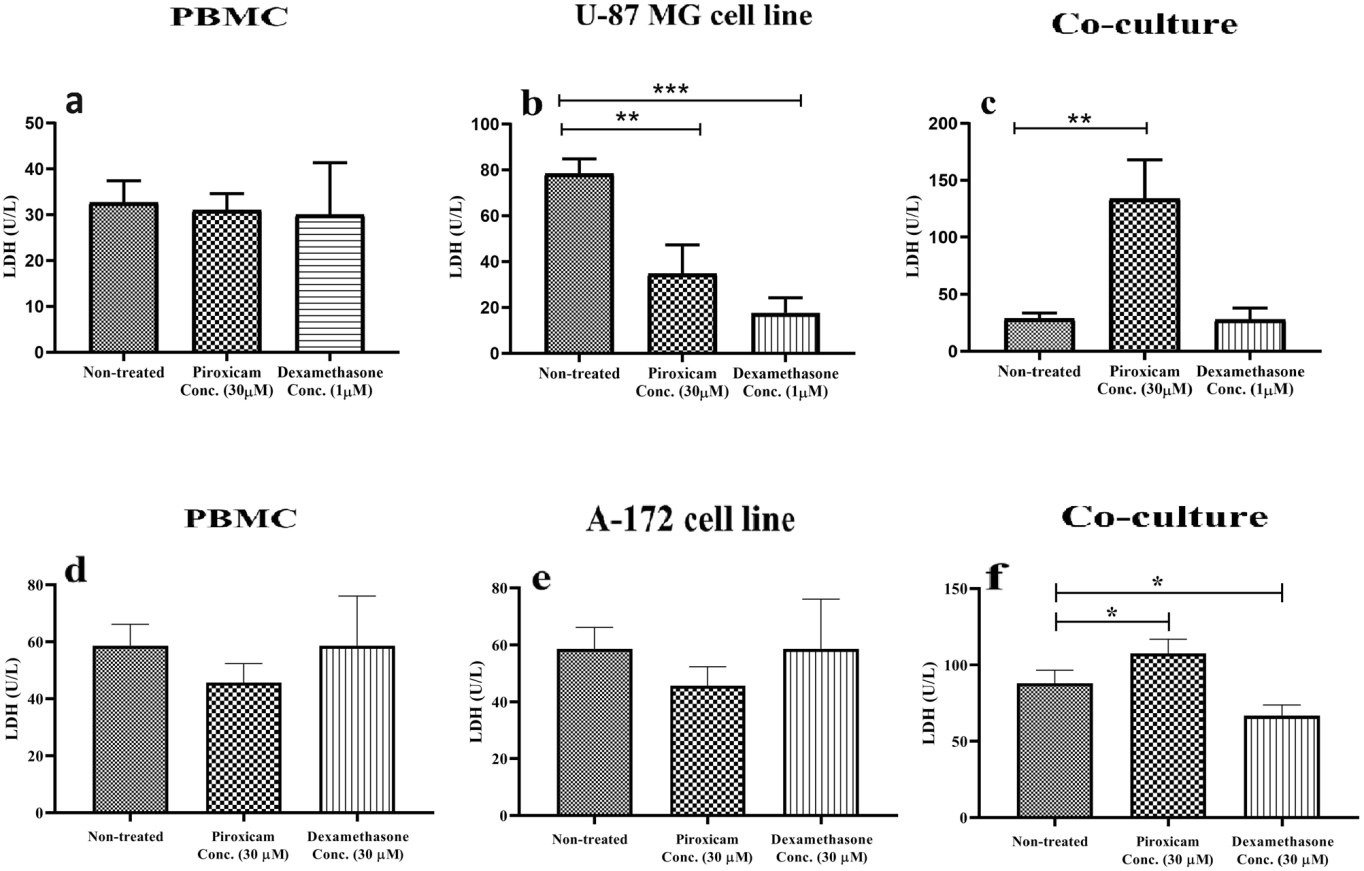
The effect of drugs on the LDH activity of T cells in PBMCs alone or in co-culture condition. The effect of dexamethasone and piroxicam on the LDH activity was compared in PBMCs cells alone (**a, d**), U-87 MG cell line (**b**), A-172 cell line (**e**), and in the co-culture condition (**c, f**) (n = 3). The graphs show the mean ± SD. Significant changes are indicated with asterisk (*p < 0.05, **p < 0.01, ***p < 0.001, ****p < 0.0001).

The role of piroxicam compared to dexamethasone on LDH activity in PBMCs was evaluated. Cells were treated with 30 µM piroxicam and 1 µM dexamethasone and stimulated with anti-CD3 and anti-CD28 antibodies. After incubation for 72 h at 37 °C, 5% CO2, and 95% humidity, supernatants were obtained, and LDH activity was measured using an autoanalyzer system. According to the data, LDH activity was not significantly different between treated and non-treated PBMCs (Fig. 7a,d, Tables 9s, 12s). Exploring the effect of drugs on LDH activity in the U-87 MG and A-172 cell lines in the same conditions revealed a considerable reduction in LDH activity of U-87 MG (Fig. 7b, Table 10s, *p* = 0.0019), but not A-172 cells (Fig. 7e, Table 13s, *p* > 0.05), in both dexamethasone and piroxicam-treated cells compared to non-treated cell line. Further evaluations in the co-culture conditions showed a substantial increment in LDH activity in the presence of piroxicam (Fig. 7c, Table 11s, *p* = 0.0013, Fig. 7f, Table 14s, *p* = 0.046), but not dexamethasone when compared to non-treated cells.

### Piroxicam partially lowered TGF-β secretion from U-87 MG and A-172 cell lines

**Figure 8:**
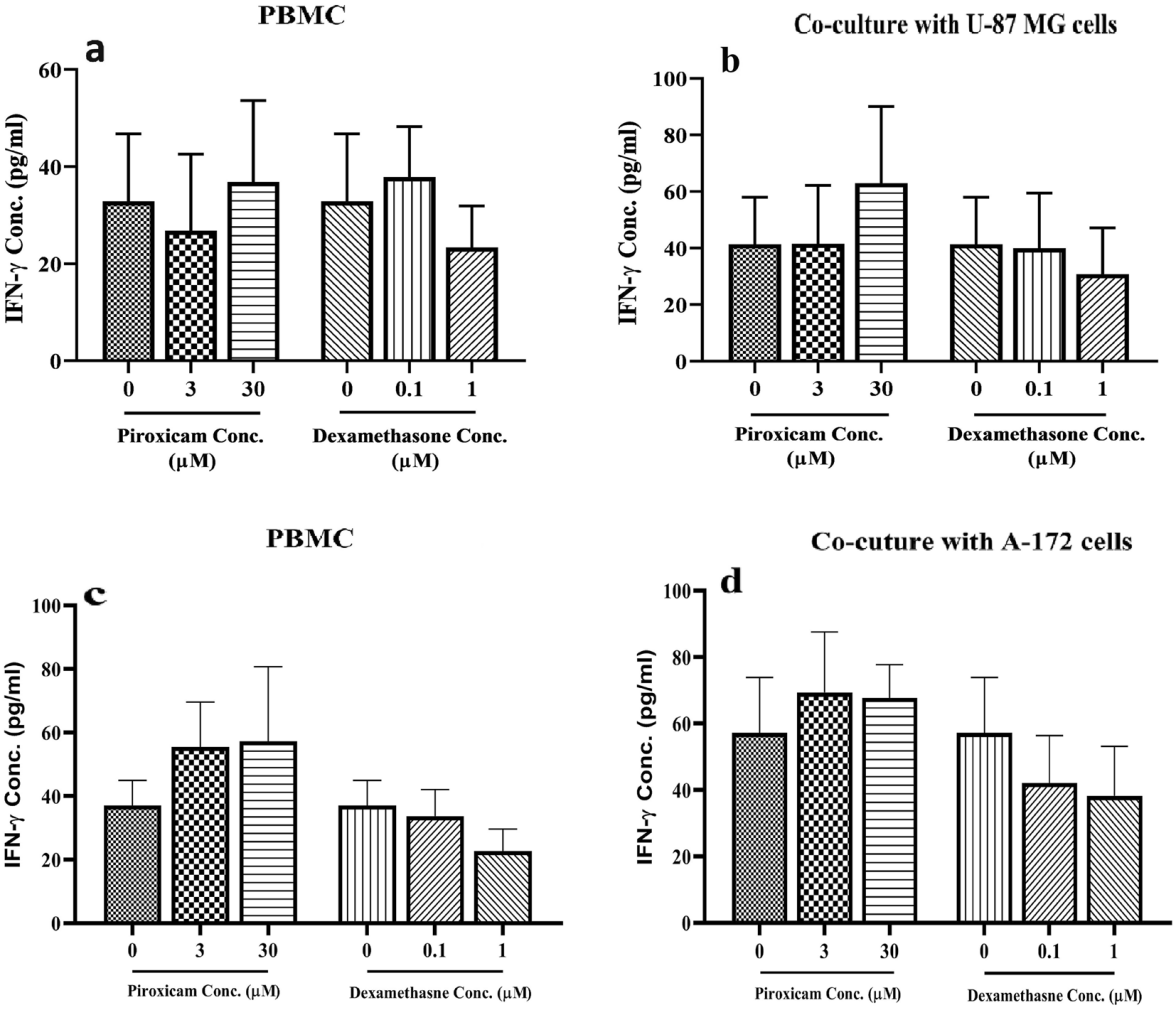
The effect of drugs on the IFN-γ levels of T cells in PBMCs alone or in co-culture condition. The effect of dexamethasone and piroxicam was measured on the IFN-γ levels of T cells in PBMCs alone (**a, c**), and in co-culture with U-87 (**b**) (n = 5), and A-172 (**d**) (n = 4) cells. The graphs show the mean ± SD. Significant changes are indicated with asterisk (*p < 0.05, **p < 0.01, ***p < 0.001, ****p < 0.0001).

**Figure 9:**
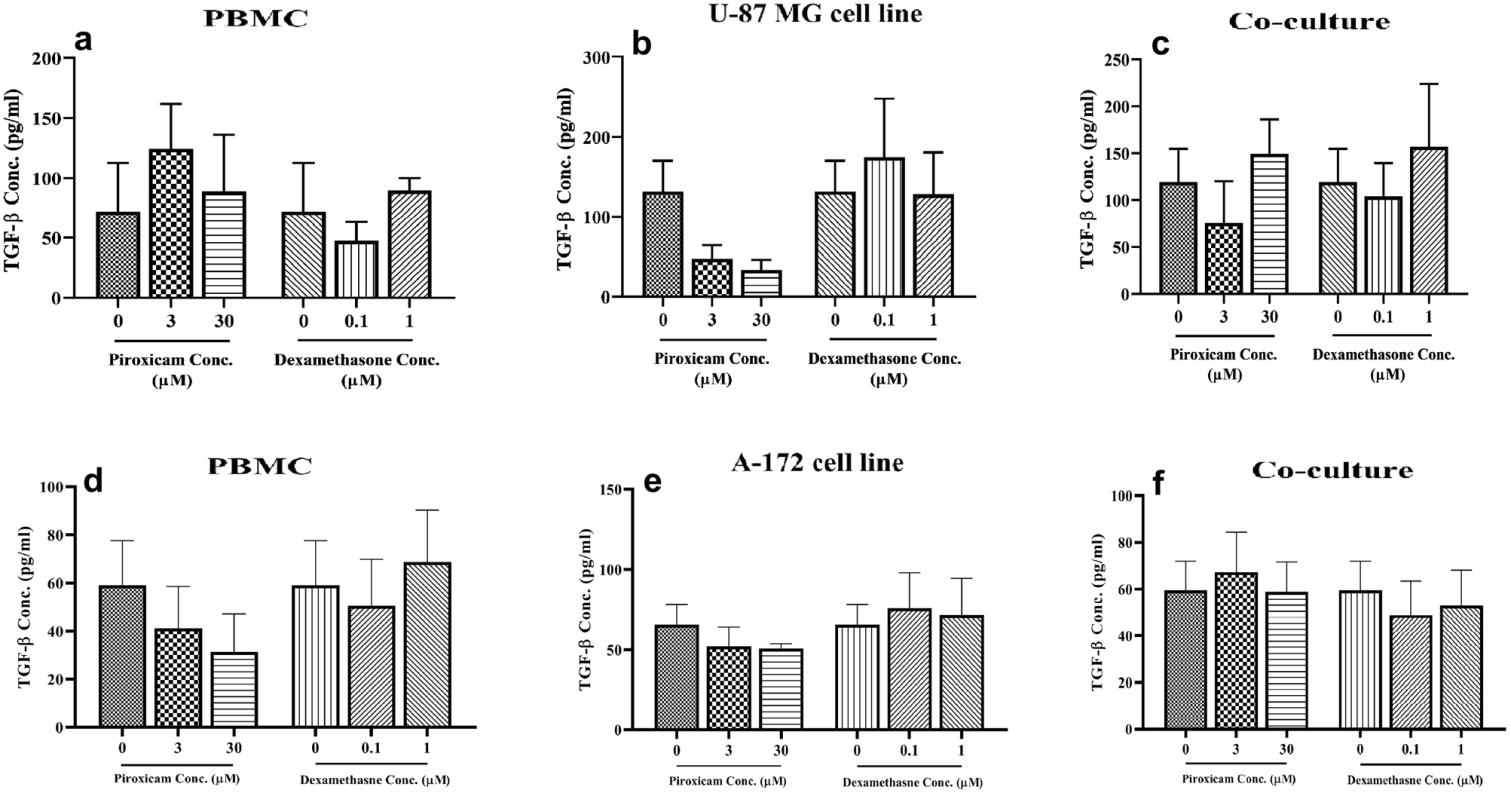
The effect of drugs on the TGF-β levels of T cells in PBMCs alone or in the co-culture condition. The effect of dexamethasone and piroxicam was measured on the TGF-β levels of T cells in PBMCs alone (**a, d**), U-87 MG cell line (**b**), A-172 cells (**e**), and in the co-culture with U-87 MG (**c**) and A-172 cells (**f**) (n = 4). The graphs show the mean ± SD. Significant changes are indicated with asterisk (*p < 0.05, **p < 0.01, ***p < 0.001, ****p < 0.0001).

The levels of IFN-γ and TGF-β produced from PBMCs were not significantly different in treated compared to non-treated cells. Two concentrations of piroxicam (3, and 30 µM) and dexamethasone (0.1, and 1 µM) were added to stimulated PBMCs alone or in co-culture with cell lines and incubated for 72 h at 37 °C, 5% CO2, and 95% humidity. No significant difference was observed between IFN-γ levels in the treated PBMCs (Fig. 8a and c, Tables 15s and 17s) and coculture (Fig. 8b and d, Tables 16s and 18s), compared to non-treated cells; however, there was a slight decline in the levels of IFN-γ in PBMCs treated with the higher concentration of dexamethasone (Fig. 8a and c). Moreover, no significant difference was observed between TGF-β levels in treated compared to non-treated cells (Fig. 9(a–f), Tab@@les S(19–24)s). However, TGF-β production was lower in the U-87 MG cell line treated with both concentrations of piroxicam, although not significant (Fig. 9b, p = 0.084).

## Discussion

Dexamethasone, widely known as an anti-inflammatory medication^30,31^, is expedient to decline chemotherapy side effects as well as the neurological symptoms in patients with brain cancers^30^. However, due to the suppressive properties of dexamethasone especially on the anti-tumor T-cell responses^32^, identifying a competent alternative seems necessary. Reports are pointing out that piroxicam, a nonselective inhibitor of the COX enzymes in the category of NSAIDs, possesses anti-edematous and anti-tumor activities besides the known anti-inflammatory actions^27^.

Tumor cells can suppress anti-tumor immune responses by secreting various factors as well as expressing surface inhibitory molecules; therefore, blocking these suppressive effects could ameliorate immune functions in the tumor microenvironment. In this in vitro study, we provided evidence that piroxicam protects peripheral blood mononuclear cells against the inhibitory effects of the U-87 MG and A-172 tumor cell lines. To the best of our knowledge, this is the first study showing the protective effect of piroxicam on PBMCs against glioblastoma tumor cells, reflecting the compensatory effect of this NSAID on the proliferation and activity of T cells in the presence of glioblastoma cell lines. In accordance, some experiments have demonstrated the protective properties of other NSAIDs in several cancers. Ferrandina et al*.* indicated that celecoxib enhanced the expression of T cell receptor (TCR)-zeta chain and thus strengthened the function of tumor-infiltrating T cells in human cervical tumors^33^. Another study demonstrated that inhibition of PGE2 production by rofecoxib led to increased proliferation and Th1 differentiation in colorectal cancer^34^. Further investigations to clarify the mechanisms of piroxicam to protect T cells could be an interesting topic for future studies.

Some previous evidence has pointed out the role of NSAIDs in sustaining the proliferative responses of T cells. Studies on indomethacin proved the positive effect of this medication on the proliferation of T cells prepared from patients with various types of cancers^35,36^. Furthermore, other NSAIDs such as diclofenac, meclofenamic acid, and ketoprofen have also exhibited immunostimulatory effects^37,38^. According to our data, while dexamethasone lowered considerably the proliferation of PBMCs, piroxicam had no negative effect on the proliferation. Moreover, cell cycle analysis revealed that dexamethasone induced the accumulation of PBMCs in the G0/G1 phase and partially limited the cell cycle progression into the S and G2/M phases. In addition, investigations on the effect of piroxicam on A-172 cell line showed a reduction in the fraction of cells in the G2/M phase. We selected two different concentrations of piroxicam and dexamethasone and compared their effects on PBMCs in both stimulated and non-stimulated conditions. The results disclosed that after stimulation of T cells with anti-CD3 and anti-CD28 antibodies, the suppressive effect of dexamethasone was more prominent, indicating that dexamethasone probably restrained T cells expansion by interfering with the TCR signaling during stimulation. Similarly, recent data have suggested that glucocorticoids can influence T-cells proliferation and their response by modulating the expression of downstream molecules involved in TCR signaling^32^. In addition, Giles et al. revealed that dexamethasone-induced cytotoxic T-lymphocyte associated protein 4 (CTLA4) expression on T cells, which compromises proliferation and IFN-γ production in T cells by blocking the CD28 signaling pathway on a glioma mouse model^39^.

We found a slight increase in SOD3 activity and TAC levels in piroxicam-treated PBMCs, although not significant. Additionally, no rise was observed in the antioxidant activity of PBMCs treated with dexamethasone. In this context, Mutsaers et.al assessed the impact of dexamethasone on the metabolism of murine-derived neural stem cells. In this study, dexamethasone downregulated 72% of the genes encoding the mitochondrial respiratory chain, and 29% of the genes related to antioxidant enzymes, with no effect on cell viability^40^. In contrast, Ortega-Martínez et.al observed that dexamethasone caused cell death in the three different astrocytoma cell lines by inducing oxidative stress^30^. The induction of oxidative stress and reduction in antioxidant activity has also been described as mechanisms for glucocorticoid-induced T-cell damage. According to these reports, dexamethasone compromises the production of antioxidants such as glutathione (GSH) and superoxide dismutase (SOD) and induces the generation of ROS in T cells; as a result, the proliferation and cytokine production decrease notably, and apoptotic cell death increase in T cells^41^**.**

Lactate dehydrogenase (LDH) is an enzyme that converts the conversion of pyruvate to lactate during the anaerobic glycolytic pathway^42^. Tumors as quickly proliferating cells prefer glycolytic pathways characterized by elevated LDH activity and lactate levels, causing poorer patient survival^43,44^. Chirasani et.al found that diclofenac, as an NSAID, downregulates the expression of the LDH-A isoenzyme, and thus limits lactate production in murine glioma cells, accompanied by a decrement in tumor cell proliferation^43^. In addition, Leidgens et.al surveyed the metabolic effects of two NSAIDs (ibuprofen and diclofenac) on human glioblastoma cell lines, U-87 MG and A172. According to their results, both agents impaired LDH-A, although treatment with ibuprofen elicited stronger effects on the enzyme activity^44^. Consistent with these data, we observed decreased LDH activity in U-87 MG cells after treatment with piroxicam. Although not statically significant, a slight decline was also observed in the LDH activity of A-172 cells. Given that high lactate levels diminished antitumor T cell responses, this finding suggested that piroxicam might counteract lactate-induced immunosuppression by limiting tumor metabolism in the tumor microenvironment. Further investigations revealed that piroxicam-treated PBMCs augmented remarkably the LDH activity, and glycolysis index, when co-cultured with both U-87 MG and A-172 cell lines, but dexamethasone did not increase the LDH activity. These data suggested that piroxicam might improve the suppressive microenvironment, induced by cell lines, and consequently, fortify T cell metabolic function, through declining LDH activity in the tumor cells. To sum up, these findings could explain a mechanism of piroxicam to protect PBMCs against the inhibitory capacity of the glioblastoma cell lines.

Recent evidence suggested that glucocorticoids polarize CD4^+^ T cell differentiation into Th2 or T regulatory cells^32^. Prenek et.al showed that in vitro treatment of mouse thymic and splenic T cells with high dose (10^–6^ M) dexamethasone elevated the expression of forkhead box P3 (Foxp3) as well as immunosuppressive cytokines, such as IL-10 and TGF-β^45^. There are conflicting data regarding the effect of NSAIDs on TGF-β production. In a study performed on acute leukemia cells, celecoxib mitigated VEGF and TGF-β levels, resulting in the inhibition of angiogenesis by tumor cells^46^. In contrast, Zhao et. al indicated that aspirin and metformin induce apoptosis in 4T1 cells through an increment in TGF-β secretion by these cells^47^. In this study, we did not find significant alteration, but when compared to the non-treated cells, a high concentration of dexamethasone alleviated IFN-γ levels in PBMCs. Moreover, although statically not significant, a high concentration of piroxicam impeded TGF-β levels in both U-87 MG and A-172 cells.

To conclude, our study adds to the growing literature on the anti-cancer properties of NSAIDs. The results of the present study showed favorable effects of piroxicam on the protection of PBMCs against inhibitory mechanisms of the U-87 MG and A-172 cell lines. In addition, a marked increase in LDH activity, as a glycolysis index, in PBMCs exposed to piroxicam, confirmed a possible role for this medication to sustain T cell function in the tumor microenvironment.

## Methods

### PBMC separation

Heparinized peripheral blood was collected from 5 healthy volunteers and PBMCs were separated using a density gradient centrifugation protocol (Ficoll paque, sigma, USA) by a standard procedure. Briefly, blood samples were diluted 1:1 with PBS and transferred into a conical tube pre-filled with Ficoll paque solution. The cells were then centrifuged at 800 × g for 20 min at room temperature, and the PBMC layer was collected. Isolated cells were counted and resuspended in RPMI-1640 medium (Capricorn Scientific, Ebsdorfergrund, Germany) supplemented with 10% heat-inactivated fetal bovine serum (FBS), 100 U/mL penicillin plus 100 μg/mL streptomycin (Gibco, Germany), and incubated at 37 °C in a humidified, 5% CO2 atmosphere. This study was approved by the Ethics Committee of Kerman University of medical sciences (98,000,781). All research was performed in accordance with relevant guidelines. Informed consent was obtained from all healthy donors.

### Cell line

Two human glioblastoma cell lines, U-87 MG (Figure 1sa) and A-172 (Figure 1sb) cells were obtained from the national cell bank of Iran (Pasteur Institute, Tehran, Iran) and cultured in RPMI-1640 medium (Capricorn Scientific, Ebsdorfergrund, Germany) supplemented with 10% heat-inactivated fetal bovine serum (FBS), 100 U/mL penicillin plus 100 μg/mL streptomycin (Gibco, Germany), under standard conditions (at 37 °C, 5% CO2 and 95% humidity). After culturing in 25 cm2 tissue flasks, the cells were trypsinized and then seeded into flat-bottomed adherent 48-well plates.

### Cell proliferation assay

The proliferative rate of PBMCs was measured by MTT (Sigma Aldrich, St Louis, USA) assay. 7 × 10^5^ PBMCs were stimulated by anti-CD3 (0.157 μg/mL, Biolegend, clone OKT3, cat. 317,302) and anti-CD28 (0.085 μg/mL, Biolegend, clone CD28.2, cat. 302,901) antibodies (Figures 2sa, 2sb) and cultured in the absence or presence of 7 × 10^4^ cell line (Figures 3sa, 3sb) (E:T ratio, 10:1). Cells were treated with different concentration of drugs (piroxicam: 0, 3, 14, and 30 µM; and dexamethasone: 0, 0.1, 0.5, 1 µM) for 72 h in 48-well plates. MTT assay was used to evaluate cell proliferation. First, 20 µl of MTT dye (0.5 mg/ml) was added to the wells and the plate was incubated at 37° for 3 h. After incubation time, MTT-containing media were removed and 100 µl dimethyl sulfoxide (DMSO, Merck, Germany) was immediately added to all wells to dissolve formazan crystals. The absorbance was measured at 570 nm using ELISA reader (StatFax 2100, USA). To calculate the protective effect of piroxicam and dexamethasone on PBMCs, the following formula (Eq. (1)) was utilized [Eq. (1)].

1 $$\% Protection = \frac{ODTcc - ODTp}{{ODCcc - ODCp}}$$

ODTcc = Absorbance of PBMCs co-cultured with cell line in the treated condition, ODTp = Absorbance of PBMCs in the treated condition, ODCcc = Absorbance of PBMCs co-cultured with cell line in non-treated condition, ODCp = Absorbance of PBMCs in non-treated condition.

### Cell cycle assay

Cell cycle phase distribution was investigated using flow cytometry. 7 × 10^5^ PBMCs were stimulated by anti-CD3 (Biolegend, clone OKT3, cat. 317,302) and anti-CD28 (Biolegend, clone CD28.2, cat. 302,901) antibodies and treated with different concentrations of drugs (piroxicam: 0, 3, 14, and 30 µM; and dexamethasone: 0, 0.1, 0.5, 1 µM) for 72 h in 48-well plates; for each cell line, 7 × 10^4^ cells were cultivated with the same concentration of drugs and the same incubation condition. Afterward, cells were centrifuged for 10 min at 800 × g and fixed using 70% pre-cooled ethanol. Cells were then washed and stained with PBS containing 50 µg/mL propidium iodide (PI) and 50 µg/mL RNase A. After incubation in the dark for 15 min at room temperature, the cells were subjected to flow cytometry (BD-Biosciences) to quantify the DNA content per cell. The data were analyzed using FlowJo software v10 (FlowJo-LLC).

### Determination of antioxidant-oxidative status

TAC and SOD3 activity were determined in the supernatant obtained from the PBMC culture. TAC was measured using the ferric reducing antioxidant power assay (FRAP) method. Both tests were performed using a commercial kit (ZellBio GmbH, Germany) according to the manufacturer’s instructions. TAC was expressed as μmol/L and SOD3 activity as U/mL.

### LDH assay

LDH was assessed in the supernatant of PBMC culture alone or the presence of cell lines using a commercial kit (Parsazmoon Co, Tehran, Iran), and sample absorbance was measured by Selectra XL autoanalyzer.

### Cytokine assay

To analyze the effect of drugs on the cytokine release from T cells, the levels of IFN-ɣ and TGF-β were measured in the culture supernatant from PBMCs, in the presence or absence of cell line (E:T ratio, 10:1), and treated with two concentrations of piroxicam (3 and 30 µM) and dexamethasone (0.1 and 1 µM). The levels of cytokines were determined using an ELISA kit (Karmania Pars Gene, Kerman, Iran) according to the manufacturer’s instruction.

### Statistical analysis

Analyses were performed using GraphPad Prism® 6.0 (GraphPad Software, San Diego, CA). Results were represented as the mean ± SD (standard deviation). After performing Shapiro–Wilk test for normality testing, One-way analysis of variance (ANOVA) (or nonparametric equivalent) test was used to compare the mean levels of parameters in different treated and non-treated groups; when significant differences were realized, Tuckey’s post hoc test was used to compare the means in pairs. Differences were considered statistically significant when *p* values were < 0.05.

## Supplementary Information

Supplementary Information.

## Data Availability

All data generated or analyzed during this study are included in the article and supplementary materials.
