# Supplementary material for: Protective potential of piroxicam on human peripheral blood mononuclear cells against the suppressive capacity of glioblastoma cell lines
Source: Sci Rep. 2022 Nov 17;12:19806. doi: 10.1038/s41598-022-24392-2 (PMC9672323; doi:10.1038/s41598-022-24392-2)
Supplement: Supplementary file 1 — Supplementary Information. [file 41598_2022_24392_MOESM1_ESM.docx]

**Original article**

**Protective potential of piroxicam on human peripheral blood mononuclear cells against the suppressive capacity of glioblastoma cell lines**

**Jahangir Abdesheikhi^1^, Farnaz Sedghy^1*^, Alireza Farsinejad^2,3^, Merat Mahmoudi^1^, Mahdi ranjkesh^1^,** **Meysam Ahmadi-Zeidabadi^4^**

^1^Department of Immunology, School of Medicine, Kerman University of Medical Sciences, Kerman, Iran

^2^Cell Therapy and Regenerative Medicine Comprehensive Center, Kerman University of Medical Sciences, Kerman, Iran

^3^Department of Hematology and Laboratory Sciences, Faculty of Allied Medical Sciences, Kerman University of Medical Sciences, Kerman, Iran

^4^Neuroscience research center, Institute of Neuropharmacology, Kerman University of Medical Sciences, Kerman, Iran

**^*^Correspondence Address:**

Farnaz Sedghy

Department of Immunology, School of Medicine, Kerman University of Medical Sciences, Kerman, Iran

Mailing Address:

Faculty of medicine, Shahid Bahonar University, Pajoohesh Sq, Kerman, Iran

Postal Code: 76169-133

Phone: +98 3433257660

Email: fsedghy@gmail.com

**Emails of authors:**

[j.abdeshaikhi@gmail.com](mailto:j.abdeshaikhi@gmail.com)

[fsedghy@gmail.com](mailto:fsedghy@gmail.com)

[farsinejad239@gmail.com](mailto:farsinejad239@gmail.com)

[merat.mahmoodi@yahoo.com](mailto:merat.mahmoodi@yahoo.com)

[Ranjkeshroudan@gmail.com](mailto:ranjkeshroudan@gmail.com)

Meysamcell@yahoo.com

**
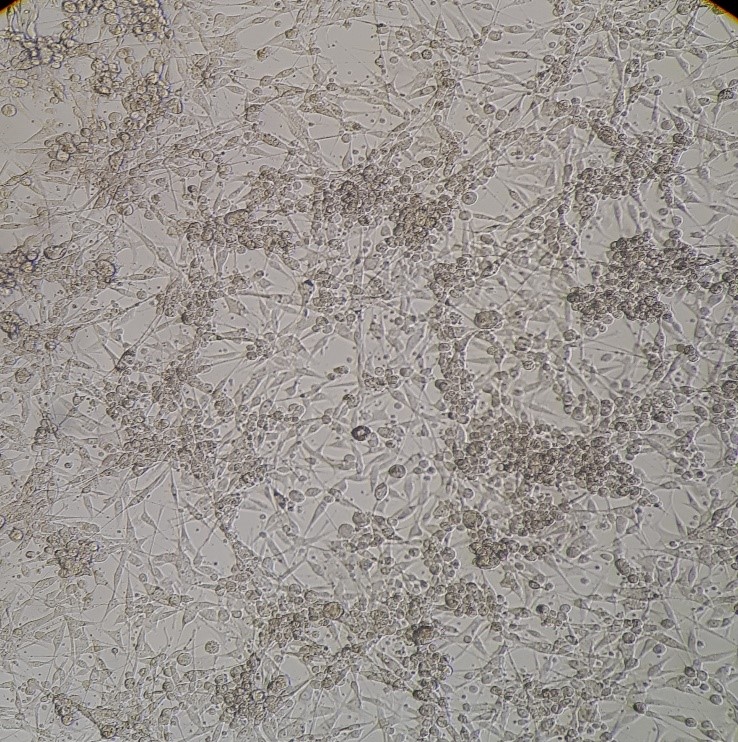

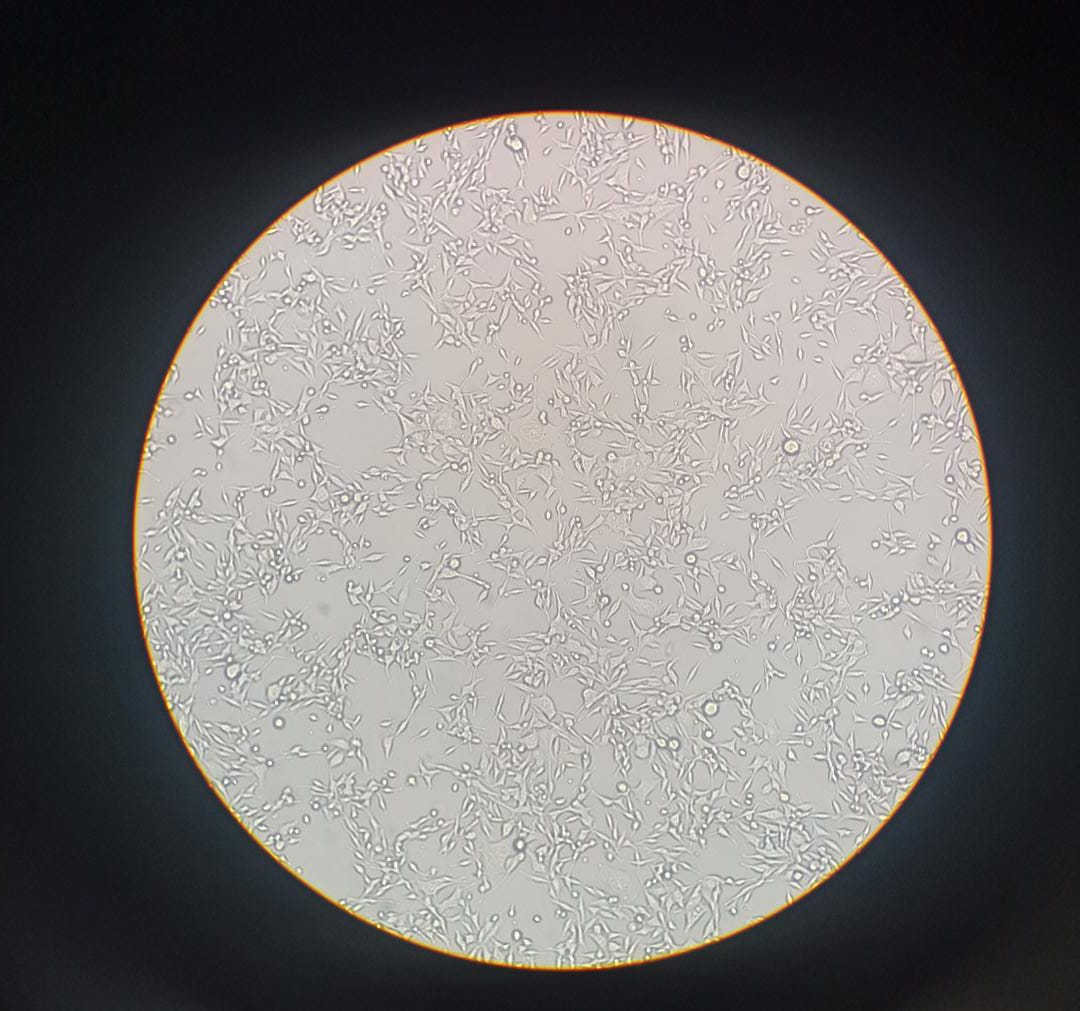
**

**b**

**a**

**Fig. 1s:** U-87 MG (a) and A-172 (b) cell line

**
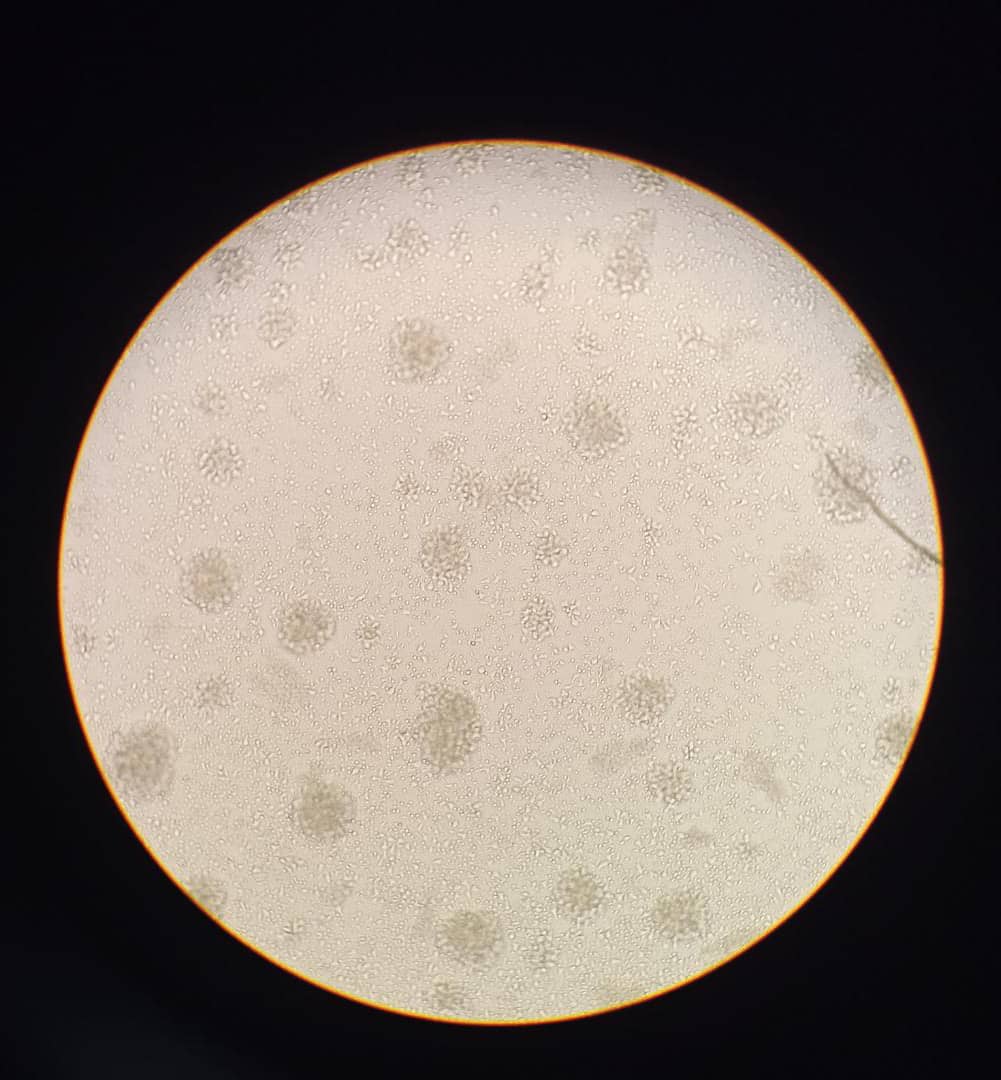

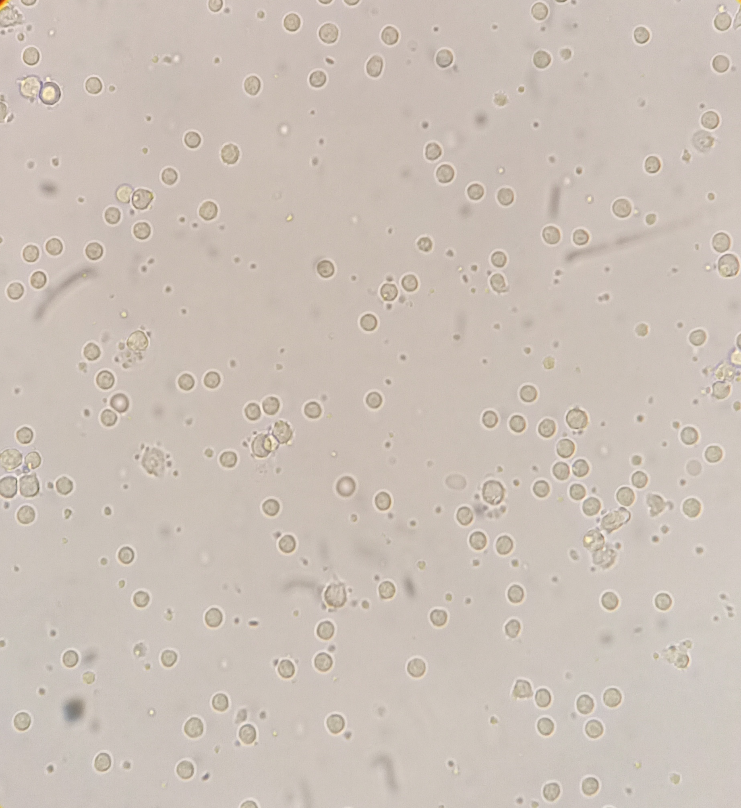
**

**a**

**b**

**Fig. 2s:** PBMCs in unstimulated (a) and stimulated (b) condition

**
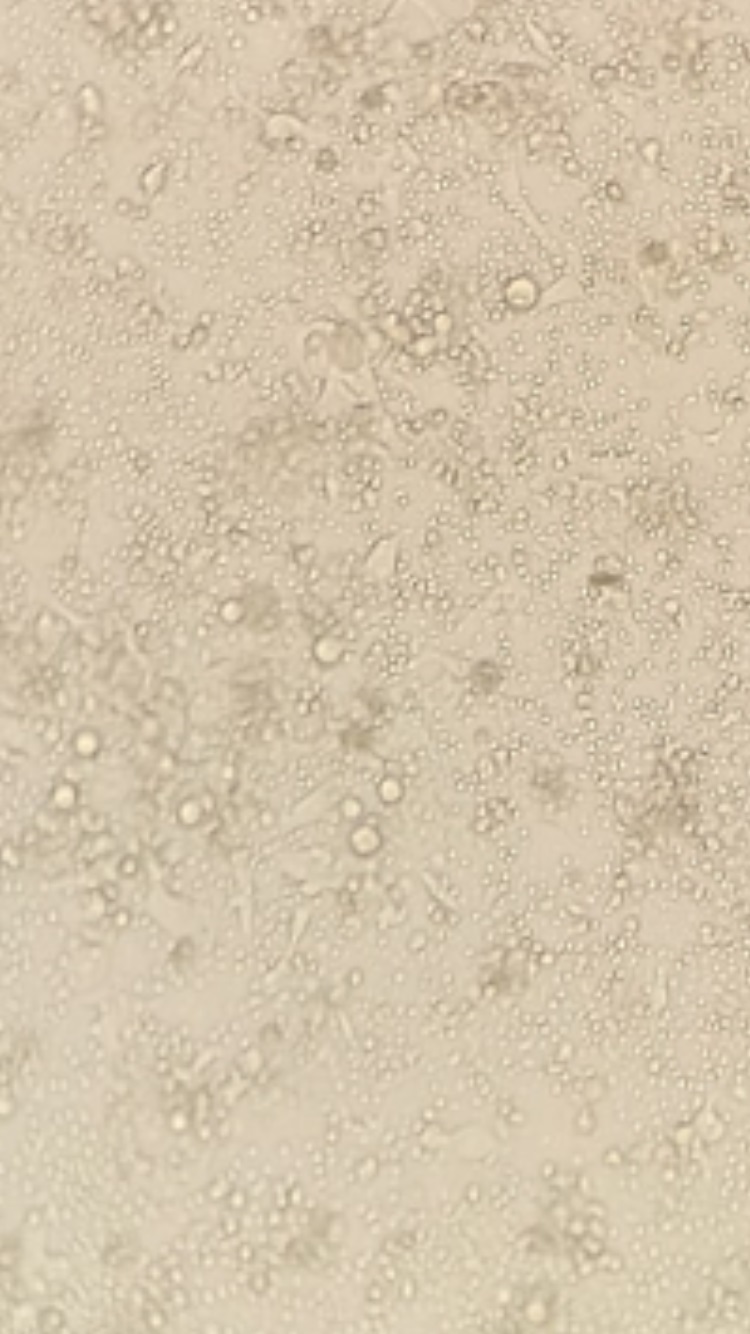

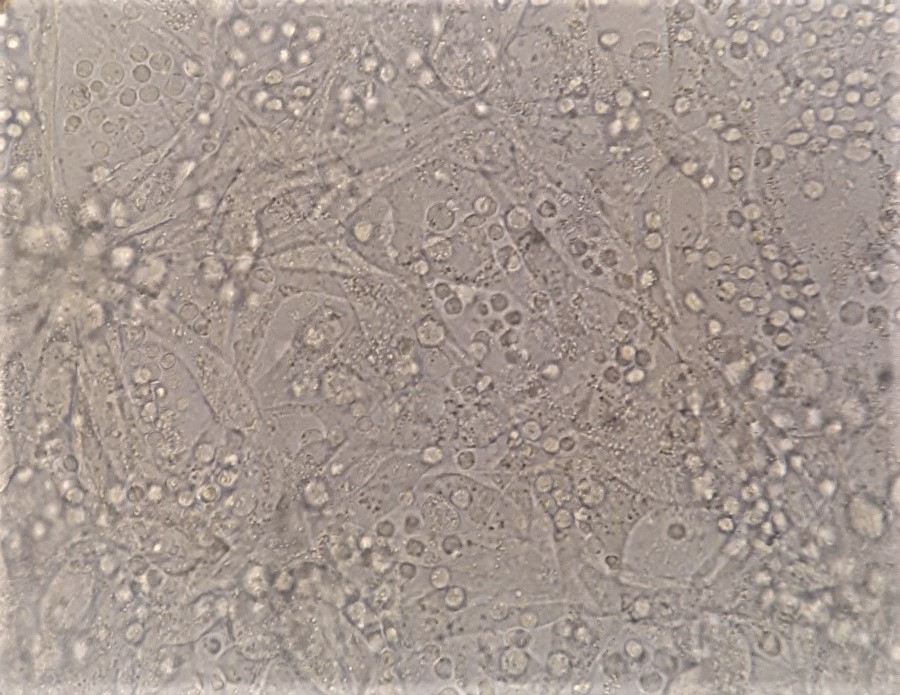
**

**a**

**b**

**Fig. 3s:** PBMCs co-cultured with U-87 MG (a) and A-172 (b) cell lines

**Table 1s**: effect of dexamethasone and piroxicam on the proliferation of T cells in PBMCs

| **Dexamethasone Con. (μM)** | **0** | **0.1** | **0.5** | **1** |
| --- | --- | --- | --- | --- |
| OD570 | 0.713 | 0.489 | 0.455 | 0.425 |
|  | 0.699 | 0.508 | 0.434 | 0.424 |
|  | 0.67 | 0.54 | 0.447 | 0.428 |

|  |  |  |  |  |  |
| --- | --- | --- | --- | --- | --- |
| **Tukey's multiple comparisons test** | **Mean Diff.** | **95.00% CI of diff.** | **Significant?** | **Summary** | **Adjusted P Value** |
| 0 vs. 0.1 | 0.1817 | 0.1352 to 0.2281 | Yes | **** | <0.0001 |
| 0 vs. 0.5 | 0.2487 | 0.2022 to 0.2951 | Yes | **** | <0.0001 |
| 0 vs. 1 | 0.2683 | 0.2219 to 0.3148 | Yes | **** | <0.0001 |
| 0.1 vs. 0.5 | 0.06700 | 0.02056 to 0.1134 | Yes | ** | 0.0074 |
| 0.1 vs. 1 | 0.08667 | 0.04022 to 0.1331 | Yes | ** | 0.0015 |
| 0.5 vs. 1 | 0.01967 | -0.02678 to 0.06611 | No | ns | 0.5568 |
| **Test details** | **Mean 1** | **Mean 2** | **Mean Diff.** | **SE of diff.** | **n** |
| 0 vs. 0.1 | 0.6940 | 0.5123 | 0.1817 | 0.01450 | 3 |
| 0 vs. 0.5 | 0.6940 | 0.4453 | 0.2487 | 0.01450 | 3 |
| 0 vs. 1 | 0.6940 | 0.4257 | 0.2683 | 0.01450 | 3 |
| 0.1 vs. 0.5 | 0.5123 | 0.4453 | 0.06700 | 0.01450 | 3 |
| 0.1 vs. 1 | 0.5123 | 0.4257 | 0.08667 | 0.01450 | 3 |
| 0.5 vs. 1 | 0.4453 | 0.4257 | 0.01967 | 0.01450 | 3 |

| **Piroxicam Con. (μM)** | **0** | **3** | **14** | **30** |
| --- | --- | --- | --- | --- |
| OD570 | 0.713 | 0.677 | 0.696 | 0.737 |
|  | 0.699 | 0.732 | 0.668 | 0.691 |
|  | 0.67 | 0.759 | 0.677 | 0.712 |

| **Dunnett's multiple comparisons test** | **Mean Diff.** | **95.00% CI of diff.** | **Significant?** | **Summary** | **Adjusted P Value** |
| --- | --- | --- | --- | --- | --- |
| **0 vs. 3** | -0.02867 | -0.09265 to 0.03532 | No | ns | 0.4701 |
| **0 vs. 14** | 0.01367 | -0.05032 to 0.07765 | No | ns | 0.8711 |
| **0 vs. 30** | -0.01933 | -0.08332 to 0.04465 | No | ns | 0.7252 |
| **Test details** | **Mean 1** | **Mean 2** | **Mean Diff.** | **SE of diff.** | **n** |
| **0 vs. 3** | 0.6940 | 0.7227 | -0.02867 | 0.02222 | 3 |
| **0 vs. 14** | 0.6940 | 0.6803 | 0.01367 | 0.02222 | 3 |
| **0 vs. 30** | 0.6940 | 0.7133 | -0.01933 | 0.02222 | 3 |

**Table 2s**: effect of dexamethasone and piroxicam on the proliferation of T cells in PBMCs in stimulated and non-stimulated conditions

| **Dexamethasone con.**  **(μM)** | **Proliferation in unstimulated condition (**OD570) | | | **Proliferation in stimulated condition (**OD570) | | |
| --- | --- | --- | --- | --- | --- | --- |
| **0** | 0.424 | 0.461 | 0.401 | 0.758 | 0.703 | 0.723 |
| **0.1** | 0.315 | 0.317 | 0.367 | 0.486 | 0.483 | 0.443 |
| **1** | 0.319 | 0.369 | 0.344 | 0.447 | 0.44 | 0.462 |

| **Tukey's multiple comparisons test** | **Mean Diff.** | **95.00% CI of diff.** | **Significant?** | **Summary** | **Adjusted P Value** |
| --- | --- | --- | --- | --- | --- |
|  |  |  |  |  |  |
| **0:Unstimulated vs. 0.1:Unstimulated** | 0.09567 | 0.02586 to 0.1655 | Yes | ** | 0.0062 |
| **0:Unstimulated vs. 1:Unstimulated** | 0.08467 | 0.01486 to 0.1545 | Yes | * | 0.0150 |
| **0:Stimulated vs. 0.1:Stimulated** | 0.2573 | 0.1875 to 0.3271 | Yes | **** | <0.0001 |
| **0:Stimulated vs. 1:Stimulated** | 0.2783 | 0.2085 to 0.3481 | Yes | **** | <0.0001 |
| **0.1:Unstimulated vs. 1:Unstimulated** | -0.01100 | -0.08080 to 0.05880 | No | ns | 0.9938 |
| **0.1:Stimulated vs. 1:Stimulated** | 0.02100 | -0.04880 to 0.09080 | No | ns | 0.9057 |

| **Test details** | **Mean 1** | **Mean 2** | **Mean Diff.** | **SE of diff.** | **n** |
| --- | --- | --- | --- | --- | --- |
|  |  |  |  |  |  |
| **0:Unstimulated vs. 0.1:Unstimulated** | 0.4287 | 0.3330 | 0.09567 | 0.02078 | 3 |
| **0:Unstimulated vs. 1:Unstimulated** | 0.4287 | 0.3440 | 0.08467 | 0.02078 | 3 |
| **0:Stimulated vs. 0.1:Stimulated** | 0.7280 | 0.4707 | 0.2573 | 0.02078 | 3 |
| **0:Stimulated vs. 1:Stimulated** | 0.7280 | 0.4497 | 0.2783 | 0.02078 | 3 |
| **0.1:Unstimulated vs. 1:Unstimulated** | 0.3330 | 0.3440 | -0.01100 | 0.02078 | 3 |
| **0.1:Stimulated vs. 1:Stimulated** | 0.4707 | 0.4497 | 0.02100 | 0.02078 | 3 |

| **Piroxicam Con. (μM)** | **Proliferation in unstimulated condition (**OD570) | | | **Proliferation in stimulated condition (**OD570) | | |
| --- | --- | --- | --- | --- | --- | --- |
| **0** | 0.424 | 0.461 | 0.401 | 0.758 | 0.703 | 0.723 |
| **3** | 0.405 | 0.382 | 0.342 | 0.627 | 0.54 | 0.484 |
| **30** | 0.578 | 0.331 | 0.458 | 0.698 | 0.649 | 0.704 |

| **Tukey's multiple comparisons test** | **Mean Diff.** | **95.00% CI of diff.** | **Significant?** | **Summary** | **Adjusted P Value** |
| --- | --- | --- | --- | --- | --- |
|  |  |  |  |  |  |
| **0:Unstimulated vs. 3:Unstimulated** | 0.05233 | -0.1357 to 0.2404 | No | ns | 0.9976 |
| **0:Unstimulated vs. 30:Unstimulated** | -0.02700 | -0.2150 to 0.1610 | No | ns | >0.9999 |
| **0:Stimulated vs. 3:Stimulated** | 0.1777 | -0.01035 to 0.3657 | No | ns | 0.0715 |
| **0:Stimulated vs. 30:Stimulated** | 0.04433 | -0.1437 to 0.2324 | No | ns | 0.9996 |
| **3:Unstimulated vs. 30:Unstimulated** | -0.07933 | -0.2674 to 0.1087 | No | ns | 0.9141 |
| **3:Stimulated vs. 30:Stimulated** | -0.1333 | -0.3214 to 0.05468 | No | ns | 0.3072 |

| **Test details** | **Mean 1** | **Mean 2** | **Mean Diff.** | **SE of diff.** | **n** |
| --- | --- | --- | --- | --- | --- |
|  |  |  |  |  |  |
| **0:Unstimulated vs. 3:Unstimulated** | 0.4287 | 0.3763 | 0.05233 | 0.05171 | 3 |
| **0:Unstimulated vs. 30:Unstimulated** | 0.4287 | 0.4557 | -0.02700 | 0.05171 | 3 |
| **0:Stimulated vs. 3:Stimulated** | 0.7280 | 0.5503 | 0.1777 | 0.05171 | 3 |
| **0:Stimulated vs. 30:Stimulated** | 0.7280 | 0.6837 | 0.04433 | 0.05171 | 3 |
| **3:Unstimulated vs. 30:Unstimulated** | 0.3763 | 0.4557 | -0.07933 | 0.05171 | 3 |
| **3:Stimulated vs. 30:Stimulated** | 0.5503 | 0.6837 | -0.1333 | 0.05171 | 3 |

**Table 3s**: the effect of drugs on the protection of T cells in PBMCs co-cultured with U-87 MG cell line

| **Drug concentration** | **Protection (%) after piroxicam treatment** | | | **Protection (%) after dexamethasone treatment** | | |
| --- | --- | --- | --- | --- | --- | --- |
| **0** | 47 | -8 | -53 | 47 | -8 | -53 |
| **1*** | 24.33333 | 34.83333 | 32.33333 | -142.333 | -144.833 | -135.833 |
| **2**** | -3.33333 | 8.166667 | 0.166667 | -159.333 | -154.333 | -164.333 |
| **3***** | -2.83333 | 9.666667 | 7.166667 | -182.167 | -176.167 | -186.667 |

| **P value** | 0.0188 |
| --- | --- |
| **P value summary** | * |
| **Significantly different (P < 0.05)?** | Yes |
| **One- or two-tailed P value?** | Two-tailed |
| **t, df** | t=3.193, df=6 |

| **Protection (%)** | **Piroxicam** | **Dexamethasone** |
| --- | --- | --- |
| **Mean** | 8.042 | -121.7 |
| **Std. Deviation** | 15.47 | 79.75 |
| **Std. Error of Mean** | 7.735 | 39.88 |
| **Lower 95% CI** | -16.57 | -248.6 |
| **Upper 95% CI** | 32.66 | 5.238 |

*Con. 1: piroxicam 3, dexamethasone 0.1. (μM) **Con. 2: piroxicam 14, dexamethasone 0.5. (μM) *Con. 3: piroxicam 30, dexamethasone 1 (μM).

**Table 4s**: the effect of drugs on the protection of T cells in PBMCs co-cultured with A-172 cell line

| **Drug concentration** | **Protection (%) after piroxicam treatment** | | | | **Protection (%) after dexamethasone treatment** | | | |
| --- | --- | --- | --- | --- | --- | --- | --- | --- |
| **0** | -10.4 | -3.47 | 5.07 | 8.8 | -10.4 | -3.47 | 5.07 | 8.8 |
| **1*** | 4.53 | 6.13 | 15.73 | 14.13 | 9.07 | 0 | 14.93 | 1.07 |
| **2**** | 12.53 | 5.6 | 14.67 | 11.47 | -3.47 | 2.93 | -8.8 | 4.53 |
| **3***** | 9.47 | 15.87 | 4.67 | 13.73 | -7.87 | 2.27 | -10.53 | -5.2 |

| **Significant?** | **P value** | **Mean of Piroxam treatment** | **Mean of Dexamethasone treatment** | **Difference** | **SE of difference** | **Adjusted P Value** |
| --- | --- | --- | --- | --- | --- | --- |
| **No** | >0.999999 | 0.000 | 0.000 | 0.000 | 6.101 | >0.999999 |
| **No** | 0.424483 | 10.13 | 6.268 | 3.863 | 4.508 | 0.668780 |
| **Yes** | 0.014826 | 11.07 | -1.203 | 12.27 | 3.628 | 0.043821 |
| **Yes** | 0.004622 | 10.94 | -5.333 | 16.27 | 3.706 | 0.018359 |

*Con. 1: piroxicam 3, dexamethasone 0.1. (μM) **Con. 2: piroxicam 14, dexamethasone 0.5. (μM) *Con. 3: piroxicam 30, dexamethasone 1 (μM).

| Cell cycle phase | G1 | | | S | | | G2/M | | |
| --- | --- | --- | --- | --- | --- | --- | --- | --- | --- |
| Non-treated | 52.09 | 51.12 | 57.43 | 20.61 | 22.63 | 18.27 | 1.95 | 2.31 | 1.66 |
| Piroxicam Con. (30 μM) | 49.69 | 45.22 | 43.11 | 11.99 | 18.16 | 15.86 | 1.81 | 1.52 | 1.42 |
| Dexamethasone Con. (1 μM) | 60.39 | 58.85 | 65.83 | 13.01 | 13.38 | 11.2 | 0.97 | 0.97 | 1.34 |

**Table 5s:** the effect of drugs on the cell cycle phase of T cells in PBMCs

| Dex | Significant? | P value | Mean of 0 | Mean of 1 | Difference | SE of difference | t ratio | df | Adjusted P Value |
| --- | --- | --- | --- | --- | --- | --- | --- | --- | --- |
| G1 | Yes | 0.047764 | 53.55 | 61.69 | -8.143 | 2.886 | 2.821 | 4.000 | 0.047764 |
| S | Yes | 0.005052 | 20.50 | 12.53 | 7.973 | 1.428 | 5.582 | 4.000 | 0.015079 |
| G2/M | Yes | 0.017338 | 1.973 | 1.093 | 0.8800 | 0.2248 | 3.914 | 4.000 | 0.034376 |

| Pirox | Significant? | P value | Mean of 0 | Mean of 30 | Difference | SE of difference | t ratio | df | Adjusted P Value |
| --- | --- | --- | --- | --- | --- | --- | --- | --- | --- |
| G1 | No | 0.052280 | 53.55 | 46.01 | 7.540 | 2.759 | 2.733 | 4.000 | 0.148782 |
| S | No | 0.078392 | 20.50 | 15.34 | 5.167 | 2.197 | 2.351 | 4.000 | 0.150639 |
| G2/M | No | 0.152959 | 1.973 | 1.583 | 0.3900 | 0.2214 | 1.761 | 4.000 | 0.152959 |

| Cell cycle phase | G1 | | | S | | | G2/M | | |
| --- | --- | --- | --- | --- | --- | --- | --- | --- | --- |
| Non-treated | 26.62 | 23.69 | 28.32 | 26.54 | 32.61 | 27.05 | 9.33 | 9.01 | 8.2 |
| Piroxicam Con. (30 μM) | 26.53 | 20.49 | 21.77 | 23.6 | 21.08 | 21.89 | 2.67 | 1.05 | 3.2 |
| Dexamethasone Con. (1 μM) | 21.91 | 24.19 | 27.14 | 25.9 | 22.79 | 28.27 | 6.42 | 5.86 | 5.02 |

**Table 6s:** the effect of drugs on the cell cycle phase of A-172 cells

|  | Significant? | P value | Mean of 0 | Mean of 1 | Difference | SE of difference | t ratio | df | Adjusted P Value |
| --- | --- | --- | --- | --- | --- | --- | --- | --- | --- |
| G1 | No | 0.426092 | 26.21 | 24.41 | 1.797 | 2.030 | 0.8851 | 4.000 | 0.491566 |
| S | No | 0.286955 | 28.73 | 25.65 | 3.080 | 2.509 | 1.227 | 4.000 | 0.491566 |
| G2/M | Yes | 0.004298 | 8.847 | 5.767 | 3.080 | 0.5278 | 5.835 | 4.000 | 0.012840 |

|  | Significant? | P value | Mean of 0 | Mean of 30 | Difference | SE of difference | t ratio | df | Adjusted P Value |
| --- | --- | --- | --- | --- | --- | --- | --- | --- | --- |
| G1 | No | 0.223898 | 26.21 | 22.93 | 3.280 | 2.281 | 1.438 | 4.000 | 0.223898 |
| S | No | 0.034702 | 28.73 | 22.19 | 6.543 | 2.081 | 3.144 | 4.000 | 0.068201 |
| G2/M | Yes | 0.000854 | 8.847 | 2.307 | 6.540 | 0.7289 | 8.972 | 4.000 | 0.002559 |

**Table 7s**: the effect of dexamethasone and piroxicam on the SOD3 activity of T cells in PBMCs

| **Group** | **Non-treated** | **Piroxicam**  **Conc. (30μM)** | **Dexamethasone**  **Conc. (1μM)** |
| --- | --- | --- | --- |
| **SOD3 activity (U/mL)** | 0.7 | 0.85 | 0.25 |
|  | 0.9 | 0.4 | 0.75 |
|  | 0.3 | 1.2 | 0.85 |

| **Dunnett's multiple comparisons test** | **Mean Diff.** | **95.00% CI of diff.** | **Significant?** | **Summary** | **Adjusted P Value** |
| --- | --- | --- | --- | --- | --- |
| **Non-treated vs. Column B** | -0.1833 | -0.9903 to 0.6236 | No | ns | 0.7512 |
| **Non-treated vs. Column C** | 0.01667 | -0.7903 to 0.8236 | No | ns | 0.9974 |
| **Test details** | **Mean 1** | **Mean 2** | **Mean Diff.** | **SE of diff.** | **n** |
| **Non-treated vs. Column B** | 0.6333 | 0.8167 | -0.1833 | 0.2819 | 3 |
| **Non-treated vs. Column C** | 0.6333 | 0.6167 | 0.01667 | 0.2819 | 3 |

**Table 8s**: the effect of dexamethasone and piroxicam on the TAC levels of T cells in PBMCs

| **Group** | **Non-treated** | **Piroxicam**  **Conc. (30μM)** | **Dexamethasone**  **Conc. (1μM)** |
| --- | --- | --- | --- |
| **TAC levels (μmol/L)** | 0.7 | 0.85 | 0.25 |
|  | 0.56 | 0.4 | 0.75 |
|  | 0.87 | 1.2 | 0.85 |

| **Dunnett's multiple comparisons test** | **Mean Diff.** | **95.00% CI of diff.** | **Significant?** | **Summary** | **Adjusted P Value** |
| --- | --- | --- | --- | --- | --- |
| **Non-treated vs. Column B** | -0.1067 | -0.8313 to 0.6180 | No | ns | 0.8819 |
| **Non-treated vs. Column C** | 0.09333 | -0.6313 to 0.8180 | No | ns | 0.9075 |
| **Test details** | Mean 1 | Mean 2 | Mean Diff. | SE of diff. | n |
| **Non-treated vs. Column B** | 0.7100 | 0.8167 | -0.1067 | 0.2531 | 3 |
| **Non-treated vs. Column C** | 0.7100 | 0.6167 | 0.09333 | 0.2531 | 3 |

**Table 9s**: the effect of drugs on the LDH activity of T cells in PBMCs

| **Group** | **Non-treated** | **Piroxicam**  **Conc. (30μM)** | **Dexamethasone**  **Conc. (1μM)** |
| --- | --- | --- | --- |
| **LDH activity (U/L)** | 31 | 34 | 25 |
|  | 38 | 27 | 43 |
|  | 29 | 32 | 22 |

| **Brown-Forsythe test** |  |  |  |  |  |
| --- | --- | --- | --- | --- | --- |
| **F (DFn, DFd)** | 0.5104 (2, 6) |  |  |  |  |
| **P value** | 0.6242 |  |  |  |  |
| **P value summary** | ns |  |  |  |  |
| **Are SDs significantly different (P < 0.05)?** | No |  |  |  |  |
| **ANOVA table** | **SS** | **DF** | **MS** | **F (DFn, DFd)** | **P value** |
| **Treatment (between columns)** | 10.89 | 2 | 5.444 | F (2, 6) = 0.09939 | P=0.9068 |
| **Residual (within columns)** | 328.7 | 6 | 54.78 |  |  |
| **Total** | 339.6 | 8 |  |  |  |

**Table 10s**: the effect of drugs on the LDH activity of U-87 MG cell line

| **Group** | **Non-treated** | **Piroxicam**  **Conc. (30μM)** | **Dexamethasone**  **Conc. (1μM)** |
| --- | --- | --- | --- |
| **LDH activity (U/L)** | 85 | 46 | 11 |
|  | 72 | 37 | 18 |
|  | 78 | 21 | 24 |

| **Dunnett's multiple comparisons test** | **Mean Diff.** | **95.00% CI of diff.** | **Significant?** | **Summary** | **Adjusted P Value** |
| --- | --- | --- | --- | --- | --- |
| **Non-treated vs. Column B** | 43.67 | 22.54 to 64.79 | Yes | ** | 0.0019 |
| **Non-treated vs. Column C** | 60.67 | 39.54 to 81.79 | Yes | *** | 0.0003 |
| **Test details** | **Mean 1** | **Mean 2** | **Mean Diff.** | **SE of diff.** | **N** |
| **Non-treated vs. Column B** | 78.33 | 34.67 | 43.67 | 7.379 | 3 |
| **Non-treated vs. Column C** | 78.33 | 17.67 | 60.67 | 7.379 | 3 |

**Table 11s:** the effect of drugs on the LDH activity in the co-culture condition (PBMC and U-87 MG cell line)

| **Group** | **Non-treated** | **Piroxicam (30μM)** | **Dexamethasone (1μM)** |
| --- | --- | --- | --- |
| **LDH activity (U/L)** | 24 | 98 | 37 |
|  | 27 | 165 | 17 |
|  | 34 | 139 | 29 |

| **Dunnett's multiple comparisons test** | **Mean Diff.** | **95.00% CI of diff.** | **Significant?** | **Summary** | **Adjusted P Value** |
| --- | --- | --- | --- | --- | --- |
| **Non-treated vs. Column B** | -105.7 | -153.7 to -57.59 | Yes | ** | 0.0013 |
| **Non-treated vs. Column C** | 0.6667 | -47.41 to 48.74 | No | Ns | 0.9988 |
| **Test details** | **Mean 1** | **Mean 2** | **Mean Diff.** | **SE of diff.** | **N** |
| **Non-treated vs. Column B** | 28.33 | 134.0 | -105.7 | 16.79 | 3 |
| **Non-treated vs. Column C** | 28.33 | 27.67 | 0.6667 | 16.79 | 3 |

**Table 12s**: the effect of drugs on the LDH activity of T cells in PBMCs

| **Group** | **Non-treated** | **Piroxicam**  **Conc. (30μM)** | **Dexamethasone**  **Conc. (1μM)** |
| --- | --- | --- | --- |
| **LDH activity (U/L)** | 20 | 20 | 15 |
|  | 27 | 22 | 19 |
|  | 35 | 26 | 18 |

| **Brown-Forsythe test** |  |  |  |  |  |
| --- | --- | --- | --- | --- | --- |
| **F (DFn, DFd)** | 1.355 (2, 6) |  |  |  |  |
| **P value** | 0.3268 |  |  |  |  |
| **P value summary** | ns |  |  |  |  |
| **Are SDs significantly different (P < 0.05)?** | No |  |  |  |  |
| **ANOVA table** | **SS** | **DF** | **MS** | **F (DFn, DFd)** | **P value** |
| **Treatment (between columns)** | 150.2 | 2 | 75.11 | F (2, 6) = 3.219 | P=0.1123 |
| **Residual (within columns)** | 140.0 | 6 | 23.33 |  |  |
| **Total** | 290.2 | 8 |  |  |  |

**Table 13s**: the effect of drugs on the LDH activity of A-172 cell line

| **Group** | **Non-treated** | **Piroxicam**  **Conc. (30μM)** | **Dexamethasone**  **Conc. (1μM)** |
| --- | --- | --- | --- |
| **LDH activity (U/L)** | 59 | 44 | 39 |
|  | 66 | 40 | 72 |
|  | 51 | 53 | 65 |

| **Dunnett's multiple comparisons test** | **Mean Diff.** | **95.00% CI of diff.** | **Significant?** | **Summary** | **Adjusted P Value** |
| --- | --- | --- | --- | --- | --- |
| **Non-treated vs. Column B** | 13.00 | -14.09 to 40.09 | No | Ns | 0.3492 |
| **Non-treated vs. Column C** | 0.000 | -27.09 to 27.09 | No | Ns | >0.9999 |
| **Test details** | **Mean 1** | **Mean 2** | **Mean Diff.** | **SE of diff.** | **n** |
| **Non-treated vs. Column B** | 58.67 | 45.67 | 13.00 | 9.463 | 3 |
| **Non-treated vs. Column C** | 58.67 | 58.67 | 0.000 | 9.463 | 3 |

**Table 14s:** the effect of drugs on the LDH activity in the co-culture condition (PBMC and A-172 cell line)

| **Group** | **Non-treated** | **Piroxicam (30μM)** | **Dexamethasone (1μM)** |
| --- | --- | --- | --- |
| **LDH activity (U/L)** | 89 | 117 | 60 |
|  | 79 | 107 | 66 |
|  | 96 | 99 | 74 |

| **Dunnett's multiple comparisons test** | **Mean Diff.** | **95.00% CI of diff.** | **Significant?** | **Summary** | **Adjusted P Value** |
| --- | --- | --- | --- | --- | --- |
| **Non-treated vs. Column B** | -19.67 | -38.93 to -0.4055 | Yes | * | 0.0463 |
| **Non-treated vs. Column C** | 21.33 | 2.072 to 40.59 | Yes | * | 0.0339 |
| **Test details** | **Mean 1** | **Mean 2** | **Mean Diff.** | **SE of diff.** | **n** |
| **Non-treated vs. Column B** | 88.00 | 107.7 | -19.67 | 6.728 | 3 |
| **Non-treated vs. Column C** | 88.00 | 66.67 | 21.33 | 6.728 | 3 |

# **Table 15s**: the effect of drugs on the IFN-γ levels of T cells in PBMCs

| **Non-treated** | **Piroxicam Con.**  **(μM)** | **3** | **30** | **Dexamethasone**  **Con. (μM)** | **0.1** | **1** |
| --- | --- | --- | --- | --- | --- | --- |
| 55.48 | **IFN-γ levels**  **(pg/ml)** | 42.48 | 27.93 | **IFN-γ levels (pg/ml)** | 46.52 | 23.21 |
| 24.55 |  | 31.35 | 60.2 |  | 37.18 | 22.71 |
| 36.57 |  | 38.8 | 44.03 |  | 50.17 | 36.57 |
| 26.8 |  | 5 | 15.7 |  | 27.3 | 21.7 |
| 21.1 |  | 16.6 | 36.4 |  | 28.1 | 12.6 |

| **Dunnett's multiple comparisons test** | **Mean Diff.** | **95.00% CI of diff.** | **Significant?** | **Summary** | **Adjusted P Value** |
| --- | --- | --- | --- | --- | --- |
| 0 vs. 3 | 6.054 | -16.98 to 29.08 | No | Ns | 0.9268 |
| 0 vs. 30 | -3.952 | -26.98 to 19.08 | No | ns | 0.9869 |
| 0 vs. 0 | 0.000 | -23.03 to 23.03 | No | ns | >0.9999 |
| 0 vs. 0.1 | -4.954 | -27.98 to 18.08 | No | ns | 0.9662 |
| 0 vs. 1 | 9.542 | -13.49 to 32.57 | No | ns | 0.6970 |
| **Test details** | **Mean 1** | **Mean 2** | **Mean Diff.** | **SE of diff.** | **n** |
| 0 vs. 3 | 32.90 | 26.85 | 6.054 | 8.544 | 5 |
| 0 vs. 30 | 32.90 | 36.85 | -3.952 | 8.544 | 5 |
| 0 vs. 0 | 32.90 | 32.90 | 0.000 | 8.544 | 5 |
| 0 vs. 0.1 | 32.90 | 37.85 | -4.954 | 8.544 | 5 |
| 0 vs. 1 | 32.90 | 23.36 | 9.542 | 8.544 | 5 |

**Table 16s**: the effect of drugs on the IFN-γ levels of T cells in co-culture condition (PBMC and U-87 MG cell line)

| **Non-treated** | **Piroxicam Con. (μM)** | **3** | **30** | **Dexamethasone Con. (μM)** | **0.1** | **1** |
| --- | --- | --- | --- | --- | --- | --- |
| 60.48 | **IFN-γ levels**  **(pg/ml)** | 50.29 | 89.9 | **IFN-γ levels**  **(pg/ml)** | 60.63 | 56.38 |
| 31.14 |  | 58.79 | 82.89 |  | 60.3 | 34.57 |
| 58.5 |  | 60.1 | 38.2 |  | 28.6 | 29.4 |
| 29.9 |  | 18.1 | 30 |  | 17.6 | 18.14 |
| 26.9 |  | 20.3 | 73.7 |  | 32.8 | 15.5 |

| **Dunnett's multiple comparisons test** | **Mean Diff.** | **95.00% CI of diff.** | **Significant?** | **Summary** | **Adjusted P Value** |
| --- | --- | --- | --- | --- | --- |
| 0 vs. 3 | -0.1320 | -33.96 to 33.70 | No | Ns | >0.9999 |
| 0 vs. 30 | -21.55 | -55.38 to 12.28 | No | Ns | 0.3189 |
| 0 vs. 0 | 0.000 | -33.83 to 33.83 | No | Ns | >0.9999 |
| 0 vs. 0.1 | 1.398 | -32.43 to 35.23 | No | Ns | 0.9999 |
| 0 vs. 1 | 10.59 | -23.24 to 44.42 | No | Ns | 0.8658 |
| **Test details** | **Mean 1** | **Mean 2** | **Mean Diff.** | **SE of diff.** | **N** |
| 0 vs. 3 | 41.38 | 41.52 | -0.1320 | 12.55 | 5 |
| 0 vs. 30 | 41.38 | 62.94 | -21.55 | 12.55 | 5 |
| 0 vs. 0 | 41.38 | 41.38 | 0.000 | 12.55 | 5 |
| 0 vs. 0.1 | 41.38 | 39.99 | 1.398 | 12.55 | 5 |
| 0 vs. 1 | 41.38 | 30.80 | 10.59 | 12.55 | 5 |

# **Table 17s**: the effect of drugs on the IFN-γ levels of T cells in PBMCs

| **Non-treated** | **Piroxicam Con.**  **(μM)** | **3** | **30** | **Dexamethasone**  **Con. (μM)** | **0.1** | **1** |
| --- | --- | --- | --- | --- | --- | --- |
| 55.48 | **IFN-γ levels**  **(pg/ml)** | 42.48 | 27.93 | **IFN-γ levels (pg/ml)** | 46.52 | 23.21 |
| 24.55 |  | 31.35 | 60.2 |  | 37.18 | 22.71 |
| 36.57 |  | 38.8 | 44.03 |  | 50.17 | 36.57 |
| 26.8 |  | 5 | 15.7 |  | 27.3 | 21.7 |
| 21.1 |  | 16.6 | 36.4 |  | 28.1 | 12.6 |

| **Dunnett's multiple comparisons test** | **Mean Diff.** | **95.00% CI of diff.** | **Significant?** | **Summary** | **Adjusted P Value** |
| --- | --- | --- | --- | --- | --- |
| 0 vs. 3 | 6.054 | -16.98 to 29.08 | No | Ns | 0.9268 |
| 0 vs. 30 | -3.952 | -26.98 to 19.08 | No | ns | 0.9869 |
| 0 vs. 0 | 0.000 | -23.03 to 23.03 | No | ns | >0.9999 |
| 0 vs. 0.1 | -4.954 | -27.98 to 18.08 | No | ns | 0.9662 |
| 0 vs. 1 | 9.542 | -13.49 to 32.57 | No | ns | 0.6970 |
| **Test details** | **Mean 1** | **Mean 2** | **Mean Diff.** | **SE of diff.** | **n** |
| 0 vs. 3 | 32.90 | 26.85 | 6.054 | 8.544 | 5 |
| 0 vs. 30 | 32.90 | 36.85 | -3.952 | 8.544 | 5 |
| 0 vs. 0 | 32.90 | 32.90 | 0.000 | 8.544 | 5 |
| 0 vs. 0.1 | 32.90 | 37.85 | -4.954 | 8.544 | 5 |
| 0 vs. 1 | 32.90 | 23.36 | 9.542 | 8.544 | 5 |

**Table 18s**: the effect of drugs on the IFN-γ levels of T cells in co-culture condition (PBMC and A-172 cell line)

| **Non-treated** | **Piroxicam Con. (μM)** | **3** | **30** | **Dexamethasone Con. (μM)** | **0.1** | **1** |
| --- | --- | --- | --- | --- | --- | --- |
| 26.95 | **IFN-γ levels**  **(pg/ml)** | 36.67 | 22.76 | **IFN-γ levels**  **(pg/ml)** | 28.9 | 27.1 |
| 46.19 |  | 55.11 | 75.5 |  | 43.09 | 14.07 |
| 38.4 |  | 59.48 | 62.13 |  | 38.03 | 20.06 |
| 36.51 |  | 70.62 | 68.22 |  | 24.69 | 29.45 |

| **Dunnett's multiple comparisons test** | **Mean Diff.** | **95.00% CI of diff.** | **Significant?** | **Summary** | **Adjusted P Value** |
| --- | --- | --- | --- | --- | --- |
| 0 vs. 3 | -18.46 | -43.66 to 6.746 | No | ns | 0.1998 |
| 0 vs. 30 | -20.14 | -45.34 to 5.064 | No | ns | 0.1446 |
| 0 vs. 0 | 0.000 | -25.20 to 25.20 | No | ns | >0.9999 |
| 0 vs. 0.1 | 3.335 | -21.87 to 28.54 | No | ns | 0.9953 |
| 0 vs. 1 | 14.34 | -10.86 to 39.55 | No | ns | 0.4049 |
| **Test details** | **Mean 1** | **Mean 2** | **Mean Diff.** | **SE of diff.** | **n** |
| 0 vs. 3 | 37.01 | 55.47 | -18.46 | 9.127 | 4 |
| 0 vs. 30 | 37.01 | 57.15 | -20.14 | 9.127 | 4 |
| 0 vs. 0 | 37.01 | 37.01 | 0.000 | 9.127 | 4 |
| 0 vs. 0.1 | 37.01 | 33.68 | 3.335 | 9.127 | 4 |
| 0 vs. 1 | 37.01 | 22.67 | 14.34 | 9.127 | 4 |

**Table 19s:** the effect of drugs on the TGF-β levels of T cells in PBMCs

| **Non-treated** | **Piroxicam Con.**  **(μM)** | **3** | **30** | **Dexamethasone**  **Con. (μM)** | **0.1** | **1** |
| --- | --- | --- | --- | --- | --- | --- |
| 97.4 | **TGF-β levels (pg/ml)** | 179.54 | 86.9 | **TGF-β levels (pg/ml)** | 50.94 | 87.77 |
| 56.35 |  | 111.74 | 151.34 |  | 33.74 | 99.4 |
| 23.1 |  | 96.5 | 36.5 |  | 68 | 95.9 |
| 112.28 |  | 110.8 | 80.6 |  | 37.9 | 76.7 |
|  |  |  |  |  |  |  |

| **Dunnett's multiple comparisons test** | **Mean Diff.** | **95.00% CI of diff.** | **Significant?** | **Summary** | **Adjusted P Value** |
| --- | --- | --- | --- | --- | --- |
| 0 vs. 3 | -52.36 | -120.2 to 15.45 | No | ns | 0.1650 |
| 0 vs. 30 | -16.55 | -84.36 to 51.26 | No | ns | 0.9382 |
| 0 vs. 0 | 0.000 | -67.81 to 67.81 | No | ns | >0.9999 |
| 0 vs. 0.1 | 24.64 | -43.17 to 92.45 | No | ns | 0.7723 |
| 0 vs. 1 | -17.66 | -85.47 to 50.15 | No | ns | 0.9216 |
| **Test details** | **Mean 1** | **Mean 2** | **Mean Diff.** | **SE of diff.** | **n** |
| 0 vs. 3 | 72.28 | 124.6 | -52.36 | 24.56 | 4 |
| 0 vs. 30 | 72.28 | 88.84 | -16.55 | 24.56 | 4 |
| 0 vs. 0 | 72.28 | 72.28 | 0.000 | 24.56 | 4 |
| 0 vs. 0.1 | 72.28 | 47.65 | 24.64 | 24.56 | 4 |
| 0 vs. 1 | 72.28 | 89.94 | -17.66 | 24.56 | 4 |

**Table 20s:** the effect of drugs on the TGF-β levels of U-87 MG cell line

| **Non-treated** | **Piroxicam Con.**  **(μM)** | **3** | **30** | **Dexamethasone**  **Con. (μM)** | **0.1** | **1** |
| --- | --- | --- | --- | --- | --- | --- |
| 176.5 | **TGF-β levels (pg/ml)** | 33.6 | 23.3 | **TGF-β levels (pg/ml)** | 187 | 188 |
| 105.4 |  | 42.3 | 30.4 |  | 241 | 89.9 |
| 113.7 |  | 66.3 | 46.8 |  | 96.9 | 107.1 |
|  |  |  |  |  |  |  |
|  |  |  |  |  |  |  |

| **Holm-Sidak's multiple comparisons test** | **Mean Diff.** | **Significant?** | **Summary** | **Adjusted P Value** |
| --- | --- | --- | --- | --- |
| **0 vs. 3** | 84.47 | No | ns | 0.1359 |
| **0 vs. 30** | 98.37 | No | ns | 0.0847 |
| **0 vs. 0** | 0.000 | No | ns | >0.9999 |
| **0 vs. 0.1** | -43.10 | No | ns | 0.5800 |
| **0 vs. 1** | 3.533 | No | ns | 0.9941 |
| **Test details** | **Mean 1** | **Mean 2** | **Mean Diff.** | **SE of diff.** |
| **0 vs. 3** | 131.9 | 47.40 | 84.47 | 35.74 |
| **0 vs. 30** | 131.9 | 33.50 | 98.37 | 35.74 |
| **0 vs. 0** | 131.9 | 131.9 | 0.000 | 35.74 |
| **0 vs. 0.1** | 131.9 | 175.0 | -43.10 | 35.74 |
| **0 vs. 1** | 131.9 | 128.3 | 3.533 | 35.74 |

**Table 21s:** the effect of drugs on the TGF-β levels of T cells in co-culture condition (PBMC and U-87 MG cell line)

| **Non-treated** | **Piroxicam Con.**  **(μM)** | **3** | **30** | **Dexamethasone**  **Con. (μM)** | **0.1** | **1** |
| --- | --- | --- | --- | --- | --- | --- |
| 153.44 | **TGF-β levels (pg/ml)** | 132.4 | 162.24 | **TGF-β levels (pg/ml)** | 74.33 | 246.14 |
| 133.44 |  | 69.8 | 178.9 |  | 75.64 | 129.84 |
| 70.9 |  | 76.4 | 160.6 |  | 146 | 88.5 |
| 120.4 |  | 24.2 | 95.8 |  | 120.6 | 163.3 |
|  |  |  |  |  |  |  |

| **Dunnett's multiple comparisons test** | **Mean Diff.** | **95.00% CI of diff.** | **Significant?** | **Summary** | **Adjusted P Value** |
| --- | --- | --- | --- | --- | --- |
| 0 vs. 3 | 43.85 | -41.63 to 129.3 | No | ns | 0.4987 |
| 0 vs. 30 | -29.84 | -115.3 to 55.64 | No | ns | 0.7967 |
| 0 vs. 0 | 0.000 | -85.48 to 85.48 | No | ns | >0.9999 |
| 0 vs. 0.1 | 15.40 | -70.08 to 100.9 | No | ns | 0.9816 |
| 0 vs. 1 | -37.40 | -122.9 to 48.08 | No | ns | 0.6366 |
| **Test details** | **Mean 1** | **Mean 2** | **Mean Diff.** | **SE of diff.** | **n** |
| 0 vs. 3 | 119.5 | 75.70 | 43.85 | 30.95 | 4 |
| 0 vs. 30 | 119.5 | 149.4 | -29.84 | 30.95 | 4 |
| 0 vs. 0 | 119.5 | 119.5 | 0.000 | 30.95 | 4 |
| 0 vs. 0.1 | 119.5 | 104.1 | 15.40 | 30.95 | 4 |
| 0 vs. 1 | 119.5 | 156.9 | -37.40 | 30.95 | 4 |

**Table 22s:** the effect of drugs on the TGF-β levels of T cells in PBMCs

| **Non-treated** | **Piroxicam Con.**  **(μM)** | **3** | **30** | **Dexamethasone**  **Con. (μM)** | **0.1** | **1** |
| --- | --- | --- | --- | --- | --- | --- |
| 47.15 | **TGF-β levels (pg/ml)** | 35.817 | 54.143 | **TGF-β levels (pg/ml)** | 45.03 | 88.46 |
| 86.18 |  | 21.09 | 24.045 |  | 32.86 | 76.9 |
| 56.22 |  | 62.73 | 18.05 |  | 77.96 | 38.03 |
| 46.39 |  | 44.7 | 28.87 |  | 46.51 | 71.33 |
|  |  |  |  |  |  |  |

| **Dunnett's multiple comparisons test** | **Mean Diff.** | **95.00% CI of diff.** | **Significant?** | **Summary** | **Adjusted P Value** |
| --- | --- | --- | --- | --- | --- |
| 0 vs. 3 | 17.90 | -18.55 to 54.35 | No | ns | 0.5376 |
| 0 vs. 30 | 27.71 | -8.745 to 64.16 | No | ns | 0.1750 |
| 0 vs. 0 | 0.000 | -36.45 to 36.45 | No | ns | >0.9999 |
| 0 vs. 0.1 | 8.395 | -28.06 to 44.85 | No | ns | 0.9504 |
| 0 vs. 1 | -9.695 | -46.15 to 26.76 | No | ns | 0.9155 |
| **Test details** | **Mean 1** | **Mean 2** | **Mean Diff.** | **SE of diff.** | **n** |
| 0 vs. 3 | 58.99 | 41.08 | 17.90 | 13.20 | 4 |
| 0 vs. 30 | 58.99 | 31.28 | 27.71 | 13.20 | 4 |
| 0 vs. 0 | 58.99 | 58.99 | 0.000 | 13.20 | 4 |
| 0 vs. 0.1 | 58.99 | 50.59 | 8.395 | 13.20 | 4 |
| 0 vs. 1 | 58.99 | 68.68 | -9.695 | 13.20 | 4 |

**Table 23s:** the effect of drugs on the TGF-β levels of A-172 cell line

| **Non-treated** | **Piroxicam Con.**  **(μM)** | **3** | **30** | **Dexamethasone**  **Con. (μM)** | **0.1** | **1** |
| --- | --- | --- | --- | --- | --- | --- |
| 58.23 | **TGF-β levels (pg/ml)** | 44.69 | 47.36 | **TGF-β levels (pg/ml)** | 48.33 | 96.01 |
| 78.2 |  | 67.02 | 51.99 |  | 102.61 | 86.01 |
| 51.5 |  | 56.34 | 49.11 |  | 75.24 | 54.34 |
| 74.04 |  | 40.43 | 54.29 |  | 77.32 | 49.95 |
|  |  |  |  |  |  |  |

| **Dunnett's multiple comparisons test** | **Mean Diff.** | **Significant?** | **Summary** | **Adjusted P Value** |
| --- | --- | --- | --- | --- |
| **0 vs. 3** | 13.37 | -17.41 to 44.15 | No | ns |
| **0 vs. 30** | 14.81 | -15.97 to 45.58 | No | ns |
| **0 vs. 0** | 0.000 | -30.78 to 30.78 | No | ns |
| **0 vs. 0.1** | -10.38 | -41.16 to 20.40 | No | ns |
| **0 vs. 1** | -6.085 | -36.86 to 24.69 | No | ns |
| **Test details** | **Mean 1** | **Mean 2** | **Mean Diff.** | **SE of diff.** |
| **0 vs. 3** | 65.49 | 52.12 | 13.37 | 11.15 |
| **0 vs. 30** | 65.49 | 50.69 | 14.81 | 11.15 |
| **0 vs. 0** | 65.49 | 65.49 | 0.000 | 11.15 |
| **0 vs. 0.1** | 65.49 | 75.88 | -10.38 | 11.15 |
| **0 vs. 1** | 65.49 | 71.58 | -6.085 | 11.15 |

**Table 24s:** the effect of drugs on the TGF-β levels of T cells in co-culture condition (PBMC and A-172 cell line)

| **Non-treated** | **Piroxicam Con.**  **(μM)** | **3** | **30** | **Dexamethasone**  **Con. (μM)** | **0.1** | **1** |
| --- | --- | --- | --- | --- | --- | --- |
| 65.01 | **TGF-β levels (pg/ml)** | 65.02 | 42.35 | **TGF-β levels (pg/ml)** | 44.19 | 67.21 |
| 50.12 |  | 89.31 | 70.2 |  | 57.6 | 33.29 |
| 74.3 |  | 47.94 | 67.41 |  | 30.64 | 49.03 |
| 48.28 |  | 66.7 | 54.88 |  | 63.08 | 62.47 |
|  |  |  |  |  |  |  |

| **Dunnett's multiple comparisons test** | **Mean Diff.** | **95.00% CI of diff.** | **Significant?** | **Summary** | **Adjusted P Value** |
| --- | --- | --- | --- | --- | --- |
| 0 vs. 3 | -7.815 | -35.47 to 19.84 | No | ns | 0.8957 |
| 0 vs. 30 | 0.7175 | -26.93 to 28.37 | No | ns | >0.9999 |
| 0 vs. 0 | 0.000 | -27.65 to 27.65 | No | ns | >0.9999 |
| 0 vs. 0.1 | 10.55 | -17.10 to 38.20 | No | ns | 0.7401 |
| 0 vs. 1 | 6.427 | -21.22 to 34.08 | No | ns | 0.9486 |
| **Test details** | **Mean 1** | **Mean 2** | **Mean Diff.** | **SE of diff.** | **n** |
| 0 vs. 3 | 59.43 | 67.24 | -7.815 | 10.01 | 4 |
| 0 vs. 30 | 59.43 | 58.71 | 0.7175 | 10.01 | 4 |
| 0 vs. 0 | 59.43 | 59.43 | 0.000 | 10.01 | 4 |
| 0 vs. 0.1 | 59.43 | 48.88 | 10.55 | 10.01 | 4 |
| 0 vs. 1 | 59.43 | 53.00 | 6.427 | 10.01 | 4 |
